# Genomic and Transcriptomic Analysis of Amoebic Gill Disease Resistance in Atlantic Salmon (*Salmo salar* L.)

**DOI:** 10.3389/fgene.2019.00068

**Published:** 2019-02-27

**Authors:** Solomon Antwi Boison, Bjarne Gjerde, Borghild Hillestad, Shokouh Makvandi-Nejad, Hooman K. Moghadam

**Affiliations:** ^1^Department of Breeding and Genetics, Nofima, Ås, Norway; ^2^Department of Genetics, SalmoBreed AS, Bergen, Norway; ^3^Department of Animal Health, Norwegian Veterinary Institute, Oslo, Norway

**Keywords:** Atlantic salmon, amoebic gill disease, AGD, transcriptome, genome-wide association study, GWAS, genomic predictions

## Abstract

Amoebic gill disease (AGD) is one of the most important parasitic diseases of farmed Atlantic salmon. It is a source of major economic loss to the industry and poses significant threats to animal welfare. Previous studies have shown that resistance against this disease has a moderate, heritable genetic component, although the genes and the genetic pathways that contribute to this process have yet to be elucidated. In this study, to identify the genetic mechanisms of AGD resistance, we first investigated the molecular signatures of AGD infection in Atlantic salmon through a challenge model, where we compared the transcriptome profiles of the naïve and infected animals. We then conducted a genome-wide association analysis with 1,333 challenged tested fish to map the AGD resistance genomic regions, supported by the results from the transcriptomic data. Further, we investigated the potential of incorporating gene expression analysis results in genomic prediction to improve prediction accuracy. Our data suggest thousands of genes have modified their expression following infection, with a significant increase in the transcription of genes with functional properties in cell adhesion and a sharp decline in the abundance of various components of the immune system genes. From the genome-wide association analysis, QTL regions on chromosomes ssa04, ssa09, and ssa13 were detected to be linked with AGD resistance. In particular, we found that QTL region on ssa04 harbors members of the cadherin gene family. These genes play a critical role in target recognition and cell adhesion. The QTL region on ssa09 also is associated with another member of the cadherin gene family, protocadherin Fat 4. The associated genetic markers on ssa13 span a large genomic region that includes interleukin-18-binding protein, a gene with function essential in inhibiting the proinflammatory effect of cytokine IL18. Incorporating gene expression information through a weighted genomic relationship matrix approach decreased genomic prediction accuracy and increased bias of prediction. Together, these findings help to improve our breeding programs and animal welfare against AGD and advance our knowledge of the genetic basis of host-pathogen interactions.

## Introduction

Amoebic gill disease (AGD), caused by the infection of the protozoan *Neoparamoeba perurans* is a primary gill disease of farmed Atlantic salmon (*Salmo salar*) around the globe (Dyková et al., 2005). The disease is also known to affect other marine aquaculture species such as rainbow trout, lumpfish, seabream, and seabass (Dyková et al., 2005; Karlsbakk et al., 2013; Haugland et al., 2017). In farmed Atlantic salmon, over the past several decades, AGD has been a significant problem in Tasmania (Taylor et al., 2007), more recently in Ireland, and it is becoming a growing concern in other salmon farming countries including Norway, Chile, Scotland, and the Faroe Islands (Oldham et al., 2016). The amoeba thrives best in seawater with warmer temperatures (12–20°C) and at salinity levels of >32 ppt (part per thousands) (Taylor et al., 2007, 2009b; Oldham et al., 2016). It is expected that an increase in the seawater temperature can result in more outbreaks of this disease.

In infected Atlantic salmon, gill surfaces are covered with white mucoid spots and gill tissues are damaged and usually look visibly pale. If the disease is left untreated, high levels of morbidity and mortality can be expected. Affected fish can be treated with freshwater or hydrogen peroxide bathing (Wynne et al., 2008b; Taylor et al., 2009b). However, the direct costs associated with the logistics of freshwater and hydrogen peroxide bathing can be significant, and also, relatively high mortalities and stress can be expected as a result of these treatments. Nonetheless, these treatment options are the most widely used approaches to manage the disease, especially since the fish gets re-infected repeatedly during the seawater phase of production.

An alternative approach to use the current treatment methods is through selection for increased resistance to AGD infection, which potentially can improve the time between the treatments and serve as a permanent or long-term solution to the problem. Several studies have reported genetic variation for AGD, where heritability estimates range from 0.12 to 0.48 (Taylor et al., 2007, 2009a; Gjerde et al., 2017; Robledo et al., 2018). A few studies have also reported that the underlying genetic architecture to AGD resistance is polygenic, with a few suggestive quantitively trait loci explaining very little of the genetic and phenotypic variations (Robledo et al., 2018). However, the accuracy of selection of breeding candidates for resistance to AGD using genomic information was higher (12–18%) than using pedigree information (Robledo et al., 2018). Selection of breeding candidates using genomic information in aquaculture populations captures the within-family variation and more accurately can predict the Mendelian sampling term compared to using pedigree-based information (Daetwyler et al., 2013).

Understanding the genetic basis of resistance or tolerance against any disease is a challenging process that requires integrated knowledge of polymorphism at the DNA level along with detailed information about the host's response at various upper molecular hierarchies such as transcriptome, proteome, methylome, metabolome, and microbiome. In particular, new advances in sequencing technologies have now made it possible to obtain accurate information about the global profile of expressed genes, to assess the transcriptional variations in host response to pathogens. Up to now, however, only a few studies, mainly by utilizing microarray technology, have investigated the transcriptome profile of Atlantic salmon in response to AGD infection (Morrison et al., 2006; Wynne et al., 2008a,b; Young et al., 2008). These studies, using a 16 or 17 K array, have identified a few hundred differentially expressed transcripts between the healthy and infected animals challenged with *N. perurans*. Their findings highlight the modifications in the expression profiles of genes such as interleukin-1 beta, with potentially critical roles in AGD pathogenesis.

In addition, although a vast number of gene expression analysis have been undertaken for various disease traits in human (e.g., Prensner et al., 2011; Costa et al., 2013), livestock (e.g., Bhuju et al., 2012), and aquaculture populations (e.g., Krasnov et al., 2016), to our knowledge very little has been done to integrate this information in genomic predictions to potentially achieve higher accuracies of selection. Such lack of integration is mainly due to relatively few samples that are used for the gene expression assessment, and those individuals are usually not part of the training population in the genomic prediction analysis. Furthermore, because results from gene expression experiments are reported on the “gene-level” (i.e., how genes are up- or down-regulated), and the animals used in genomic prediction are genotyped on SNP arrays that have not necessarily been obtained as part of the differentially expressed transcript analysis, it is not straightforward on how to combine these two sources of information.

We propose that because markers on SNP arrays can be mapped directly to the genome, we can give different weights (i.e., assign higher importance) to markers that are located within or close to up- or down-regulated genes, identified from gene expression analysis, in genomic prediction models. This approach of using external information (e.g., results from gene expression analysis) to weight markers differently in genomic prediction is not new but have mainly been tested using results from genome-wide association studies (GWAS) (de Los Campos et al., 2013; Ni et al., 2017). For instance, de Los Campos et al. (2013), in studying type-2 diabetes in humans, reported an increase in predictive ability when using −*log*_10_*p* values from a GWAS in genomic best linear unbiased predictions (GBLUP). However, Ni et al. (2017) did not find higher predictive power in layer chickens using −*log*_10_*p* values as weights compared to ordinary GBLUP. Contrary to using external information as weights (e.g., GWASes) to build genomic relationships matrices for GBLUP, allele substitution effects obtained from the same dataset can also be used as weights to build genomic relationship matrices in GBLUP (Su et al., 2014; Zhang et al., 2015, 2016). Such approaches have been shown to result in increased prediction accuracy of genomic predictions.

In this study, to assist the identification and elucidation of molecular mechanisms underlying genetic resistance to AGD, we performed whole transcriptome profiling of naïve and infected animals and further integrated this information with polymorphism data from ~1,330 fish across thousands of genetic markers. We specifically focused on the phenotypes collected during the second infection, as there exist low genetic correlations between the first and subsequent encounters with *N. perurans* (average *r*_*g*_ = 0.24) (Kube et al., 2012). On the other hand, the estimated genetic correlations between the second and all later re-infections are high (~*r*_*g*_ = 0.8) (Kube et al., 2012). Therefore, in a field setting, where a fish can continuously get infected with this amoeba, it is more important and cost-effective to identify the genetic basis of resistance following the initial infection. Here we aimed to (i) investigate and identify genes and genetic networks that had modified their regulation in response to infection (ii) quantify the genetic variation of AGD resistance (iii) identify the genetic mechanisms of AGD resistance by conducting genome-wide association and gene expression analysis and (iv) to explore the potential of combining results from the gene expression analysis with SNP information to increase the accuracy of genomic selection for AGD resistance in Atlantic salmon.

## Materials and Methods

### SalmoBreed Population and Challenge Tests

#### Year-Class 2015

In 2015, 1,672 PIT-tagged (passive integrated transponder) smolts from 100 SalmoBreed year-class 2015 breeding nucleus families, the offspring of 50 sires and 100 dams (~17 fish per family), were transported from Nofima Sunndalsøra to the challenge facility VESO Vikan (Namsos). After arrival, the fish were kept on 12°C brackish water (15 ± 2%) and 24:00 light regime. Further, after the initial acclimatization, the fish were reared on full salinity water (31 ppt) and challenged with an *N. perurans* isolate (VESO Vikan ref. no. 2014.10.15NO). On a weekly basis, 30 fish were gill scored until an average gill score of 2.4 was reached at day 17 post-challenge, where all fish were scored and then treated with freshwater three consecutive times. The scorings were based on the criteria previously described by Taylor et al. (2009a,b). According to this scoring, the level of infection can range from 0 to 5. A score of 0 indicates that the gills are clear from the amoeba. A score of 1 or 2 is assigned if the gills have one or up to 2–3 small whitish spots of amoeba, respectively. A score of 3, 4, or 5 indicates that up to 20, 50, or more than 50% of the gill is covered with the amoeba. The fish were treated with freshwater for 3 h at 21, 31, and 41 days post challenge. After the freshwater treatments, all fish were re-challenged, and the gills were again monitored on a weekly basis until an average gill score of 2.8 was reached at day 24. All surviving fish were then scored before the termination of the trial.

#### Year-Class 2016

In 2016, we challenged fish from SalmoBreed year-class 2016 at Industrilaboratoriet (ILAB) challenge facility in Bergen. In total, 2,435 PIT-tagged smolts from 277 full-sib families (~9 fish per full-sib family) were transported from SalmoBreed Lønningdal breeding station to ILAB. After the initial acclimatization, the fish were challenged with an *N. perurans* isolate in full salinity water (34 ppt) with 1,000 amoebas per liter. Fish were monitored regularly for disease progression and mortalities. After approximately 2 weeks, the fish had an average gill score of 2 and were treated with freshwater for 5 h. The fish were then held at 25 ppt salinity over 5 days before being re-challenged with 500 amoebas per liter at 34 ppt. After approximately 2 weeks, when the fish had reached an average gill score of 2, all animals were scored, weighed, and the experiment was terminated. The samples and the scores from the second-gill scoring were used for further genotyping and genomic data analyses. To better assess the severity of AGD infection, the ILAB has optimized a scoring system, suggested initially by Taylor et al. (2009a,b). According to this scoring scheme, the 4-gill arches on each side of the head are scored on both sides using the scale of Taylor et al. (2009a,b), resulting in 16 independent scores. While the minimum score in this system is 0, ([*gill score*]^*^16 [*number of gills*] = 0), the maximum score is calculated as 5^*^16 = 80. The sum of these scores across all gill arches was used as the phenotype for each fish. Both challenge trials were approved by the Norwegian Animal Research Authority (NARA), and were conducted under regulations controlling experiments and procedures in live animals in Norway (the Animal Welfare Act of December 20th 1974, No 73, chapter VI sections 20-22355 and the Regulation on Animal Experimentation of January 15th 1996).

### Transcriptome Sequencing, Alignment, and Differential Gene Expression Assessment

To understand the host genetic response to AGD infection, from the animals challenged in 2015, representing 30 different SalmoBreed families, we collected the second left anterior gills from 6 naïve individuals (before the challenge test), 6 fish after the first infection and 24 fish by the termination of the test and immersed the tissues in RNALater (Ambion) for transcriptome analysis (Supplementary Figure 1). Sampling a higher number of animals with various degrees of clinical symptoms against AGD during the second challenge was aimed to increase the power of identifying genes and genetic networks central for improved resistance against this disease following the first infection. Total RNA was extracted using RNeasy Plus mini kit (Qiagen). The RNA integrity and size distribution were assessed using Bioanalyzer 2100 (Agilent Technologies). All RNA had RIN values (RNA Integrity Number) >8. Preparation of the mRNA libraries and sequencing transcripts were performed by the Norwegian High-Throughput Sequencing Centre (https://www.sequencing.uio.no/) using standard protocols (https://support.illumina.com/content/dam/illumina-support/documents/documentation/chemistry_documentation/samplepreps_truseq/truseqrna/truseq-rna-sample-prep-v2-guide-15026495-f.pdf). Samples were sequenced on an Illumina HiSeq 4000 platform as strand-specific, paired-end (PE) 150 bp reads. All the raw sequences have been deposited in the NCBI Short Read Archive (SRA) under the accession number PRJNA509327.

Following read quality assessment (www.bioinformatics.babraham.ac.uk/projects/fastqc/), removing sequencing adapters and trimming low quality bases (Bolger et al., 2014), the remaining sequences were aligned to the salmon genome assembly ICSASG_v2 using TopHat (v.2.0.13) (Trapnell et al., 2009) and reads with more than a single hit were discarded. The uniquely aligned sequences were then fed to Cufflinks (Trapnell et al., 2010, 2012) to generate transcriptome assemblies for each sequenced sample and all merged by Cuffmerge to construct a single gene transfer file. Expression data were normalized via the median of the geometric means of fragment counts across all samples, where relative expressions are expressed as fragments per kilobase of exon per million mapped reads (FPKM) values. Cuffdiff was then used to estimate the expression abundances of the assembled genes and transcripts and to test for differential levels of expression between phenotypic groups. Genes or transcripts with >1.5-fold difference in expression and corrected *p*-values (FDR adjusted) of <0.05 were assigned as differentially expressed (DE).

### Genotyping and Genotype Quality Assessment

From the challenge test performed at the ILAB in 2016, 1,585 individuals, representing 136 families, were selected to be genotyped on a custom-made 55 K Affymetrix Axiom array, developed by Nofima in 2016 in collaboration with SalmoBreed and Marine Harvest (NOFSAL03). DNA extraction and genotyping were performed by IdentiGEN Ltd. (https://identigen.com/; Dublin, Ireland). In total 1,335 fish passed the initial quality control during DNA extraction and the SNP calling steps of the Affymetrix axiom analysis suite software. Additional genotype and sample quality checks were undertaken with PLINK v1.9 (Chang et al., 2015). We discarded samples and SNPs with call rates <90%. Furthermore, SNPs with Hardy Weinberg *p*-value (Fisher's exact test) <10^−10^ and those with minor allele frequency <1% were removed. Lastly, samples with very low or high heterozygosity rate (heterozygosity rate ≤0.25 and ≥0.45) were also removed from the dataset. After the quality checks, the final data consisted of 53,109 SNPs and 1,333 samples. It should be noted that although all the animals from year-class 2015 were also genotyped (AROS Applied Biotechnology, Aarhus, Denmark), the data were unfortunately unusable due to problems associated with the correct sample ID assignment.

### Variance Components Estimation and Genomic Relationship Matrices

Variance components were estimated using phenotype information from the genotyped fish of the 2016 year-class. The following linear mixed animal model was fitted using ASReml v4 (Gilmour et al., 2009) as follows:

y=Xb+Tm+Zc+Zg+e

where *y* is the vector of AGD scores; *X, T*, and *Z* are design matrices assigning phenotype to the scoring person, tanks (fish were kept in two tanks during the experimentation period), common environmental effect of full-sibs prior to tagging and genomic breeding values (GEBV), respectively; *b* is the effect of scoring person (two scoring persons in total); *m* is the effect of tank; *c* is the effect common to full-sibs other than additive genetics; *g* is the vector of GEBVs, and *e* is the vector of residuals. The effect common to full-sibs and the residual term is assumed to follow a normal distribution $c\sim N\left(0,I\sigma_{c}^{2}\right)$ and $e\sim N\left(0,I\sigma_{e}^{2}\right)$, where *I* is an identity matrix and $\sigma_{c}^{2}$ and $\sigma_{e}^{2}$ are the variances for effects common to full-sibs and residual. GEBVs were assumed to follow a normal distribution $g\sim N\left(0,G\sigma_{g}^{2}\right)$, where *G* is the genomic relationship matrix computed using different approaches (described below) and $\sigma_{g}^{2}$ is the additive genetic variance for AGD scores. The analysis was also undertaken with a similar model in which the *G* matrix was replaced with the numerator relationship matrix and *Zg* with *Za* where *a* was the vector of additive genetic breeding values. All variance components were estimated using restricted maximum likelihood (REML).

Two different types of G matrices were constructed in this study to allow for incorporating gene expression information from differential gene expression analysis in genomic prediction. The first genomic relationship matrix (*G*_*ord*_) was constructed as follows:

$$\begin{array}{l} G_{ord}=\frac{WW^{\prime }}{2\sum_{i=1}^{Nsnp}p_{i}\left(1-p_{i}\right)} \end{array}$$

where *W* is a centered (−2*p*) matrix of marker genotypes that were coded as 0 for homozygote AA, 1 for the heterozygote (AB or BA) and 2 for homozygote BB. *p*_*i*_ is the allele frequency of the B allele, and *Nsnp* represents the total number of markers used in the analysis.

The second G matrix was constructed using information from the DE gene analysis. The *log*_2_Δ*F* values from the DE analysis was used as weights in the G matrix. In the DE analysis, the *log*_2_Δ*F* values are obtained for genes, and therefore markers on the SNP array were mapped to their corresponding gene location. We assigned the *log*_2_Δ*F* value to markers that were *n* kilobase pairs (kb) left or right of the genes start and end positions. Since not all markers are located within or near to a gene, two groups (close to a gene or not) of markers were generated. The genomic relationship matrix (*G*_*DE*_) was then computed as *G*_*DE*_ = λ*G*_*genic*_ + (1 − λ)*G*_*ord*_. *G*_*genic*_ had the following construction:

$$\begin{array}{l} G_{genic}=\left[\frac{W_{DE}D{W_{DE}}^{\prime }}{2\sum_{i=1}^{Nsnp_{DE}}{p_{i}}_{DE}\left(1-{p_{i}}_{DE}\right)}\right] \end{array}$$

where *G*_*ord*_ is as described before except that markers that were not mapped into genic regions were used. *W*_*DE*_, *p*_*i*_*DE*__, *Nsnp*_*DE*_ are as described for *G*_*ord*_ matrix except that markers from the genic regions were used to construct *G*_*genic*_. *D* is a diagonal matrix of weights which was based on the $log_{2}\Delta F/\bar{\left(log_{2}\Delta F\right)}$ of the differentially expressed transcriptome analysis. Although the *log*_2_Δ*F* from the DE analysis was used in this study, −*log*_10_*p* values obtained for each gene from the DE analysis could also be used. The script to compute the genomic relationship matrices *G*_*ord*_ and *G*_*DE*_ is available at https://github.com/soloboan/DEbased_Gmatrix. The λ parameter determines how much of the genic matrix constitute *G*_*DE*_. Just as Zhang et al. (2015) and Ni et al. (2017), λ was determined using a cross-validation scheme in the training population by searching through ranges of values from 0.1 to 1. When λ = 0, *G*_*DE*_ = *G*_*ord*_.

### Accuracy and Scale (Bias) of GEBVs

Genomic prediction of gill-scores was undertaken with the same model as detailed under the variance component estimation section. A random sample of 1,000 of the total 1,333 fish was used as training animals while the remaining fish were used as validation points. This approach was replicated 20 times and with the prediction accuracy and bias computed for each replicate. Prediction accuracy was computed for each replicate as:

$$Accuracy\;\left(r_{corr}\right)=\;\frac{\rho (GEBV,\;y_{adj})}{\sqrt{h^{2}}}$$

where ρ is the Pearson moment correlation coefficient, *GEBV* is the estimated genomic breeding values, *y*_*adj*_ is the adjusted phenotype (*y*_*adj*_ = *y* − *Xb* − *Tm*), and *h*^2^ is the heritability of the trait when λ = 0.

The scale of prediction was estimated by the regression coefficient of *y*_*adj*_ on GEBV. A regression coefficient of 1 indicates no bias in EBVs, while a value higher or <1 is indicative of deflated and inflated GEBVs, respectively. The accuracy and bias of predation results are presented as the means and standard deviations over the 20 replicates.

### Scenarios Used for the Genomic Prediction

The scenarios considered were based on the proximity of markers to the significant DE genes and the level of significance of the genes based on the DE analysis. The scenarios are presented below:

- Markers that were 25 kb of a moderately significant DE genes (*p* ≤ 2.4 × 10^−3^);
- Markers that were 100 kb of a moderately significant DE genes (*p* ≤ 2.4 × 10^−3^);
- Markers that were 25 kb of a highly significant DE genes (*p* ≤ 1.0 × 10^−4^);
- Markers that were 100 kb of a highly significant DE genes (*p* ≤ 1.0 × 10^−4^);

For scenarios i, ii, iii, and iv, the number of markers used to construct *G*_*DE*_ were 864, 2,317, 354, and 941. Variance components, heritability, prediction accuracies, and biases are presented for all these scenarios.

### Genome-Wide Association Study

GWAS was performed using the model described earlier, except that marker effects were also fitted. The model was fitted in GCTA v2 (Yang et al., 2011) as follows:

$$\begin{array}{l} y=Xb+Tm+W\alpha +Zg+e \end{array}$$

where *y, X, b, T, Z, g*, and *e* have been described earlier; *W* is the incidence matrix for marker genotypes, and α is the allele substitution effect of the SNP. Markers were considered genome- or chromosome-wide significant when they crossed the Bonferroni threshold *p* ≤ 9.41 × 10^−7^ and *p* ≤ 2.73 × 10^−5^.

## Results

### Challenge Testing and Comparative Transcriptomic Analyses of 2015 Year-Class

**Figure 1:**
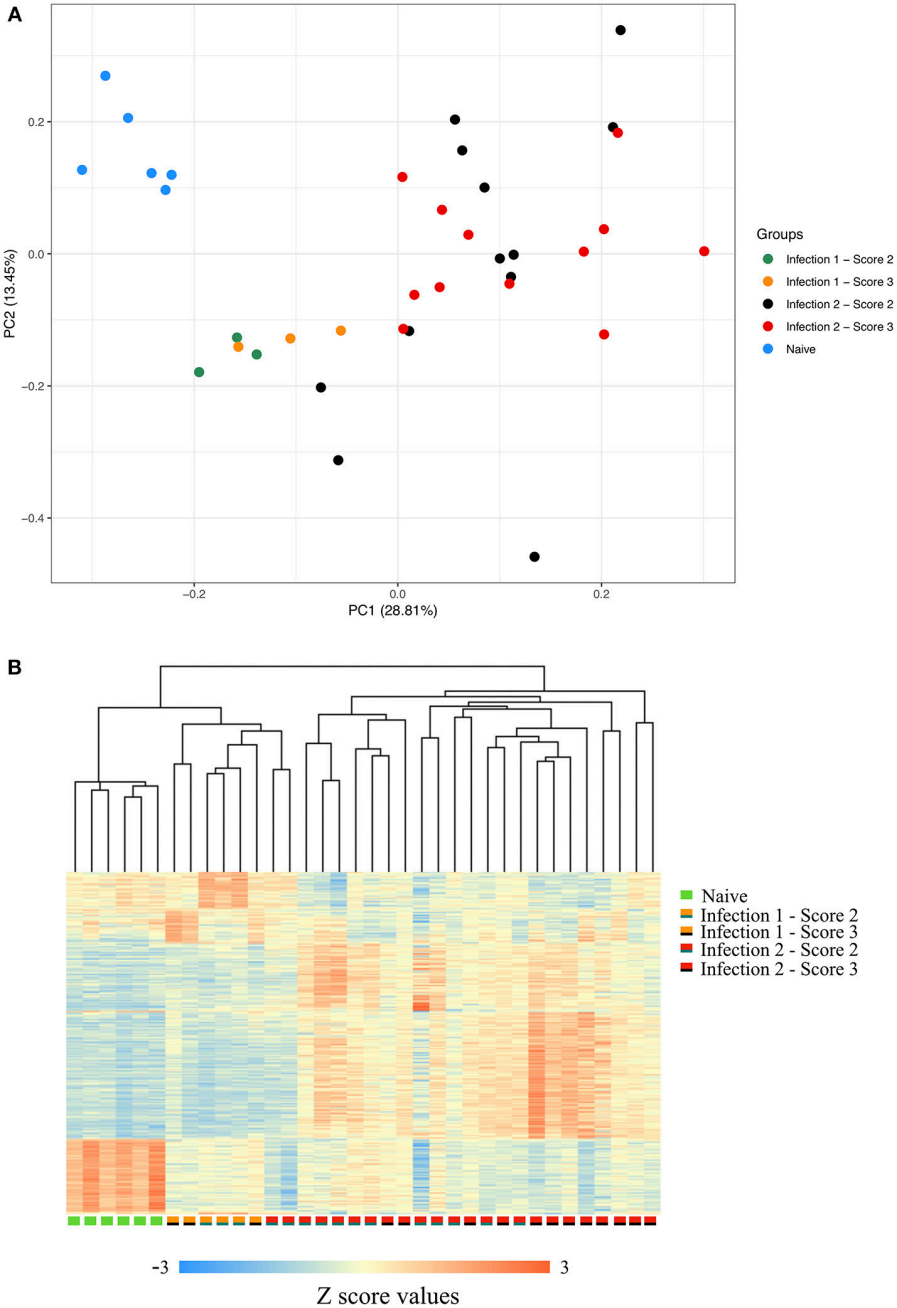
**(A)** Principle component of gene expression. **(B)** Clustering of the gene expression data between the naïve, animals during the first infection with scores 2 or 3 and animals during the second infection with scores 2 or 3. The scorings are based on the scheme outlined by Taylor et al. (2009a,b).

Following filtering the low-quality sequences and discarding reads with multiple hits against the genome, on average, we obtained more than 13 million uniquely mapped PE reads per individual. Comparative assessment of the transcriptome profiles between the three sampling stages indicated that the host's response to the amoeba infection was positively correlated with the duration at which an animal had encountered the parasite (Supplementary Figure 2). In total, we identified 887, 2,107, and 1,145 genes with significant changes in their expression between the naïve and the first, naïve and the second and the first and the second infections, respectively. The principal component analysis (PCA) of the gene expression data along with the clustering of the differentially expressed transcripts, further showed a clear distinction between the transcriptomic profiles of the host's response during each of the different stages of the challenge (Figures 1A,B). As shown in the PCA plot, the animals' global transcripts during the second infection had a higher degree of divergence and variation compared to the other two sampling stages (Figure 1A). Such higher divergence in gene expression is possibly an indication of considerable inter-individual diversity in the genes and the genetic networks that become activated following the amoeba re-infection.

**Figure 2:**
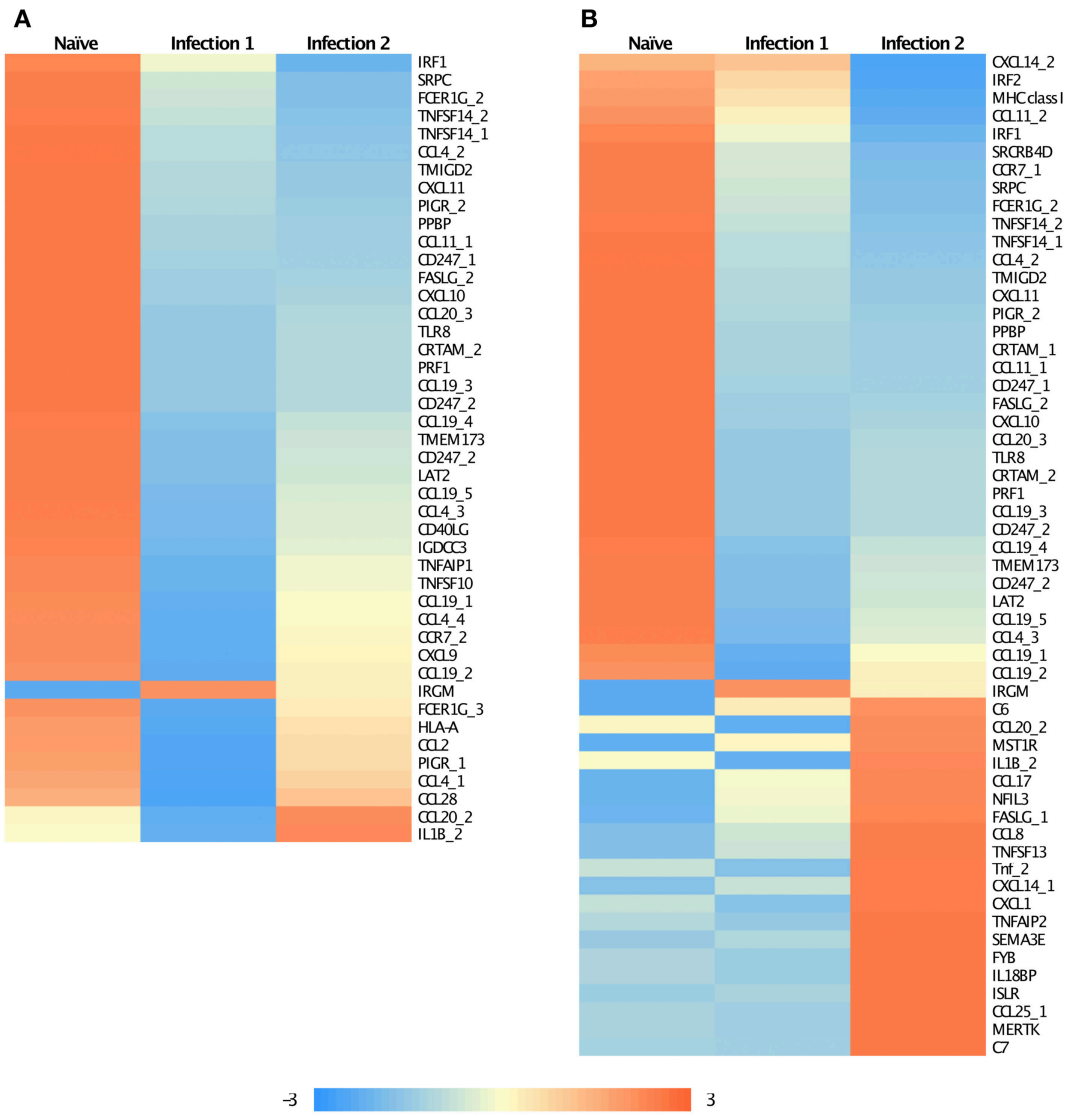
Heatmap of the standardized FPKM gene expression, showing relative levels of abundance of differentially regulated immune related transcripts between **(A)** Naïve and animals during the first infection and **(B)** Naïve and animals during the second infection. Numbers following an underscore represent putative duplicated genes.

Enrichment analysis of Gene Ontology (GO) associated terms among the DE genes showed a significant reduction in the transcription of different components of immune-related genes following both infections compared to the naïve animals (Supplementary Figures 3a,b). These genes are mainly involved in the innate immunity of fish and particularly function in immunoregulatory, inflammatory responses, and chemotaxis (Figures 2A,B). For instance, many of these genes encode chemokine ligands such as chemokine CC motif ligand 4 (CCL4), ligand 19 (CCL19), chemokine CXC motif ligand 9 (CXCL9), and ligand 10 (CXCL10) (Figures 2A,B). For some of these genes, we found similar changes in their expression profiles among the paralogues and/or the homeologues. For example, we detected multiple copies of CCL19 on ssa01, ssa11, ssa15, and ssa24 to have significantly down-regulated their expressions. Chromosome ssa01 shares duplicated regions with chromosome ssa11 and similarly, ssa15 has ancestral segments in common with ssa24 (Lien et al., 2016).

It is interesting to note that some of the down-regulated genes in the primary infection were slightly up-regulated during the subsequent encounter with amoeba (Figure 2A), even though they still retained lower expression levels compared to the naïve animals. Majority of these genes encode for chemokines and are involved in recruiting T- and B-cells in association with antigen-presenting cells such as dendritic cells (DCs). A similar observation has previously been reported for *Leishmania*, the parasitic agents, causative of leishmaniasis (Liu et al., 2009). It was shown that the parasite could influence the host's early immune response by modulating DCs function in a way to inhibit their antigen-presenting role, and consequently, affect the T-cell response. Here, a similar explanation can also be proposed, although, further investigations are required.

We are also reporting some immune genes that have significantly elevated their transcription levels in infected animals. Primary innate immune response, following the first infection, was initiated by the up-regulation of a single gene, immunity-related GTPase M (IRGM; Figure 2A). IRGM plays a significant role in the clearance of acute protozoan infection via autophagy canonical pathway (GO:0006914) (Feng et al., 2009). Moreover, it has been suggested that the product of this gene may also be involved in adhesion and mobility of interferon (IFN)-gamma-activated macrophages (Henry et al., 2009). However, the expression of this gene was reduced in the secondary infection as the immune response started to shift from innate to adaptive. Following the re-infection, modification in the expression of immune-related genes involved in pathways associated with chemotaxis, inflammatory response, and cytokine production was identified, of which about 20 genes had significantly up-regulated their expression compared to the naïve fish (Figure 2B; Supplementary Figures 3c,d). We found evidence of putative paralogous or homeologous copies of CXCL14 on ssa05 and ssa09, apoptosis antigen ligand (FASLG) on ssa20 and ssa23 and CCL20 on ssa03 and ssa15 to show alternative patterns of expression between the duplicates (Figure 2B). Such an alternative profile in expression between duplicated gene copies might be a suggestion of possible neofunctionalization of these genes following the whole genome duplication (Amoutzias et al., 2010).

**Figure 3:**
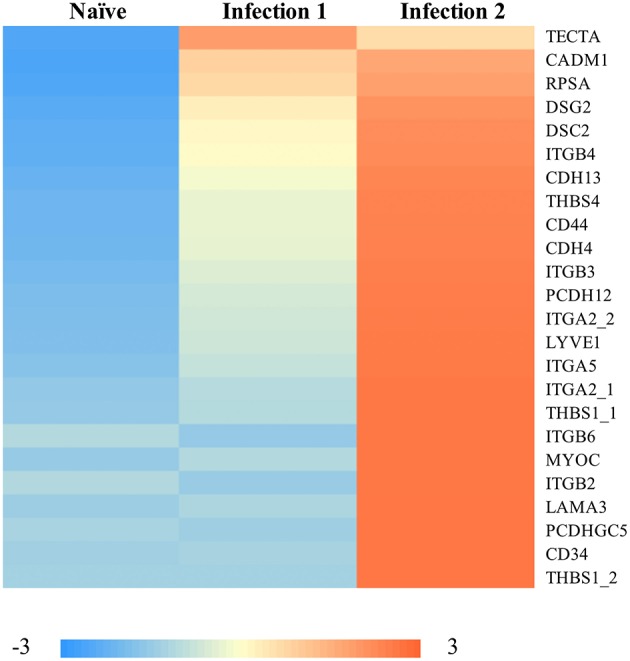
Heatmap of the standardized FPKM gene expression, showing relative levels of abundance of differentially regulated transcripts with functional properties in “cell-adhesion” between naïve and animals during the 2nd infection. Numbers following an underscore represent putative duplicated genes.

Some of the up-regulated genes such as CCL8 and CCL20 (Figure 2B) are associated with the recruitment of T-cells, B-cells, natural killer cells (NK), and DCs to the inflammatory sites (e.g., Jensen and Gad, 2010). However, the most enriched groups of up-regulated genes in animals during the second infection are genes with functional properties related to cell and biological adhesion (Figure 3; Supplementary Figure 3c). For instance, we identified different members of the integrin receptor family, belonging to both alpha and beta subunits, to have elevated their levels of transcription (e.g., ITGA2, ITGA5, ITGB6). These cell surface glycoproteins are not only crucial for mediating cell-cell and cell-extracellular matrix adhesion (Giancotti and Ruoslahti, 1999), but also play significant roles in the recruitment and activation of immune cells (Evans et al., 2009). Also, same as before, among these genes we further detected signatures of up-regulation of putative homeologes located in duplicated chromosomal regions. The duplicated copies of integrin alpha 2 (ITGA2) on ssa01 and ssa13 as well as copies of thrombospondin-1 (THBS1) on the homeologous segments of ssa01 and ssa09 showed significant up-regulation during the second infection.

Comparing the transcriptomics of animals in the second challenge, with different levels of infection (i.e., score 2 vs. score 3), we detected signatures of 289 differentially expressed genes, with 206 having significantly higher expression in animals with score 3. In particular, among the transcripts with elevated levels of expression in animals with score 3, we found enrichment of genes with functional properties in cell adhesion. These genes included 1. Thrombospondin-2, a member of thrombospondins gene family of calcium-binding glycoproteins, with roles in regulation of angiogenesis, connective tissue organization and immune response (Bentley and Adams, 2010), 2. Integrin alpha-8 with functions as explained above, 3. Brevican core protein, a proteoglycan that is involved in cell adhesion, movement, and neuronal plasticity (Gill et al., 2012), 4. Thrombospondin-1, an adhesive glycoprotein associated with angiogenesis, motility, and cell growth (Lawler, 2002), and 5. Tenascin C, an extracellular matrix glycoprotein with a critical role in cell signaling in particular in response to inflammation and tissue injury (Midwood and Orend, 2009). Further, we also found signatures of many long-noncoding RNA (lncRNA) with different transcription patterns between the two scoring phenotypes. We expect these transcripts to be involved in the transcription, translation and regulation of at least some of the differentially expressed genes reported in this study (Carrieri et al., 2012).

On the other hand, among the transcripts with significantly higher expression in animals with less severe infection, we identified a few immune-related genes. These genes included: perforin-1, a glycoprotein with expression mainly limited to the natural killer and CD8^+^ T-cells, which is released to the target cells upon cell-cell contact (Voskoboinik et al., 2015). Ectonucleotide pyrophosphatase/phosphodiesterase family member 2, encoded by *ENPP2* gene, also a glycoprotein, with functions in both physiological and pathological pathways that include inflammation and oncogenesis processes (Farquhar et al., 2017). And finally, chemokine ligand 20 (CCL20), a principal regulator of both innate and acquired immunity that acts as a chemoattractant for immature dendritic cells, memory T lymphocytes, and naïve B-cells (Schutyser et al., 2003).

### Challenge Test, Phenotypic Scores, and Genotyping of 2016 Year-Class

The phenotypic distribution of AGD scores for the 1,333 genotyped animals of the 2016 year-class following the second challenge is presented in Supplementary Figure 4. The minimum, maximum, average and median scores were 0, 29, 5.79 (SD = 4.16), and 5.0, respectively. About 99.0% of the fish received a score of ≤ 20. When using the scoring system of Taylor et al. (2009a,b), a gill score value of 3 and above directly implies how severe a fish has been infected and treatment commences when the average score exceeds 2. However, although the new scoring system is detailed, it might be difficult to find the ideal score that reflects infection severity.

### Pedigree and Genomic Variance Components

Moderate heritability estimate of AGD infection was obtained using pedigree (0.32 ± 0.15) and genomic (0.28 ± 0.05) information (Table 1). Therefore, the estimate of heritability using genomic information was about 12.5% lower than that of pedigree. The shared environmental variance was estimated to be about 4.2–4.7% of the total phenotypic variance and 13.1–16.8% of the genetic variance.

**Table 1:** Estimates of variance components and heritability from pedigree and genomic information.

**Parameters**	**Pedigree**	**Genomic**
$\sigma_{g}^{2}$	4.380 (2.168)	3.821 (0.804)
$\sigma_{c}^{2}$	0.575 (0.958)	0.643 (0.390)
$\sigma_{e}^{2}$	8.812 (1.174)	9.200 (0.504)
$\sigma_{p}^{2}$	13.767 (0.638)	13.665 (0.627)
*h*^*2*^	0.318 (0.153)	0.280 (0.053)
*c*^*2*^	0.042 (0.069)	0.047 (0.028)

*$\sigma_{g}^{\text{2}}$, genetic variance; $\sigma_{c}^{\text{2}}$, common environmental variance; $\sigma_{e}^{2}$, residual variance; $\sigma_{p}^{2}$, phenotypic variance; h^2^, heritability*.

### Genome-Wide Association Results

**Figure 4:**
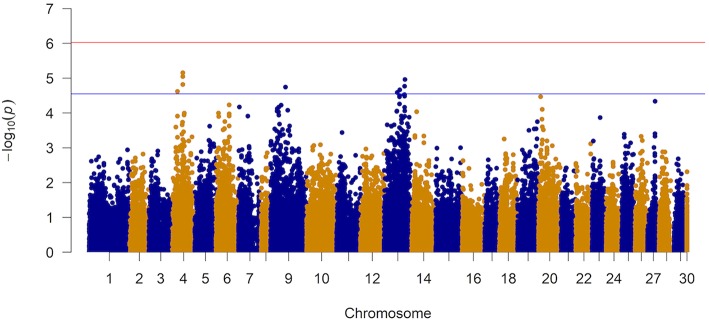
Manhattan plot from the genome-wide marker analysis of resistance to AGD. The red and blue represent Bonferroni and chromosome-wide significance thresholds.

The results of the GWAS are presented in Figure 4 and Table 2. Twelve markers exceeded the chromosome-wide significant threshold of *p* ≤ 2.73 × 10^−5^ threshold. The markers are located on chromosomes 4, 9, and 13. Allele frequencies for the significant markers ranged from 6 to 46%. In addition, the proportion of genetic and phenotypic variance captured by each marker was <5.5 and <2.0%, respectively. Markers AX-97870670, AX-87625334, AX-87645824, and AX-97870670 on ssa04 were in high linkage disequilibrium (LD; *r*^2^ = 0.98). These markers fall within a region of the chromosome that contains various members of the cadherin gene family and at least one of these genes were identified to be differentially expressed between the naïve and animals at the second infection. AX-88308327 on ssa09 is located in a genomic segment containing protocadherin Fat 4 gene, a member of the Fat cadherin subfamily with function in influencing planar cell polarity and Hippo signaling (Sadeqzadeh et al., 2014). Markers on ssa13 were also found to be in high LD (*r*^2^ = 0.98). Four out of six associated markers on ssa13 are in relatively close proximity to each other, spanning a genomic segment of approximately three million bp. Within this region, we are reporting signatures of at least 11 genes with different transcription profiles between sampling stages, which included interleukin-18-binding protein, a key gene with an important product for inhibiting the proinflammatory effect of cytokine IL18 (Dinarello et al., 2013).

**Table 2:** Summary statistics of QTL regions associated with AGD infection.

**CHR**	**SNP**	**BP**	**AA/AB/BB**	**MAF**	**ASE (α)**	**−*logP***	***V*_*m*_*g***	***V*_*m*_*p***
4	AX-97870670	39584257	2/166/1165	0.064	1.429	5.155	5.076	1.769
4	AX-87625334	39589437	2/167/1161	0.064	1.408	5.050	4.928	1.717
13	AX-87090633	81271722	24/281/1028	0.123	1.087	4.964	5.289	1.843
4	AX-87645824	39590212	2/165/1166	0.064	1.381	4.819	4.741	1.652
13	AX-96143022	80037333	99/576/657	0.291	−0.751	4.769	4.828	1.683
9	AX-88308327	58205845	7/220/1104	0.088	−1.187	4.746	4.692	1.635
13	AX-88260877	61211215	79/596/637	0.287	−0.768	4.666	5.008	1.745
4	AX-96497078	18773593	274/684/375	0.463	−0.698	4.620	5.026	1.752
13	AX-96340731	51418953	207/695/400	0.426	−0.723	4.589	5.304	1.848
13	AX-98318366	80039614	90/580/661	0.286	−0.739	4.526	4.627	1.613
13	AX-87232771	78068947	36/436/852	0.192	−0.853	4.524	4.684	1.632

*CHR, chromosome; BP, base pairs; MAF, minor allele frequency; AA/AB/BB, genotype counts; ASE, Allele substitution effect; V_m_g, proportion of total genomic variance explained by SNP ($2pq\alpha^{2}/\sigma_{g}^{2}=$3.28) and V_m_p, proportion of total phenotypic variance explained by SNP $\left(2pq\alpha^{2}/\left[\sigma_{p}^{2}=13.63\right]\right)$*.

The Q-Q plot of the observed and expected *p*-values is presented in Supplementary Figure 5. Based on the observed and expected test statistics, the inflation factor of 1.23 was obtained which indicated a slight inflation of the observed p-values. When the association analysis was performed with the top 5 eigenvectors (they explained ~10% of the variation in relationships among animals) from a PCA, the inflation factor did not change significantly.

### Variance Component Estimate With Varying DE Information

**Figure 5:**
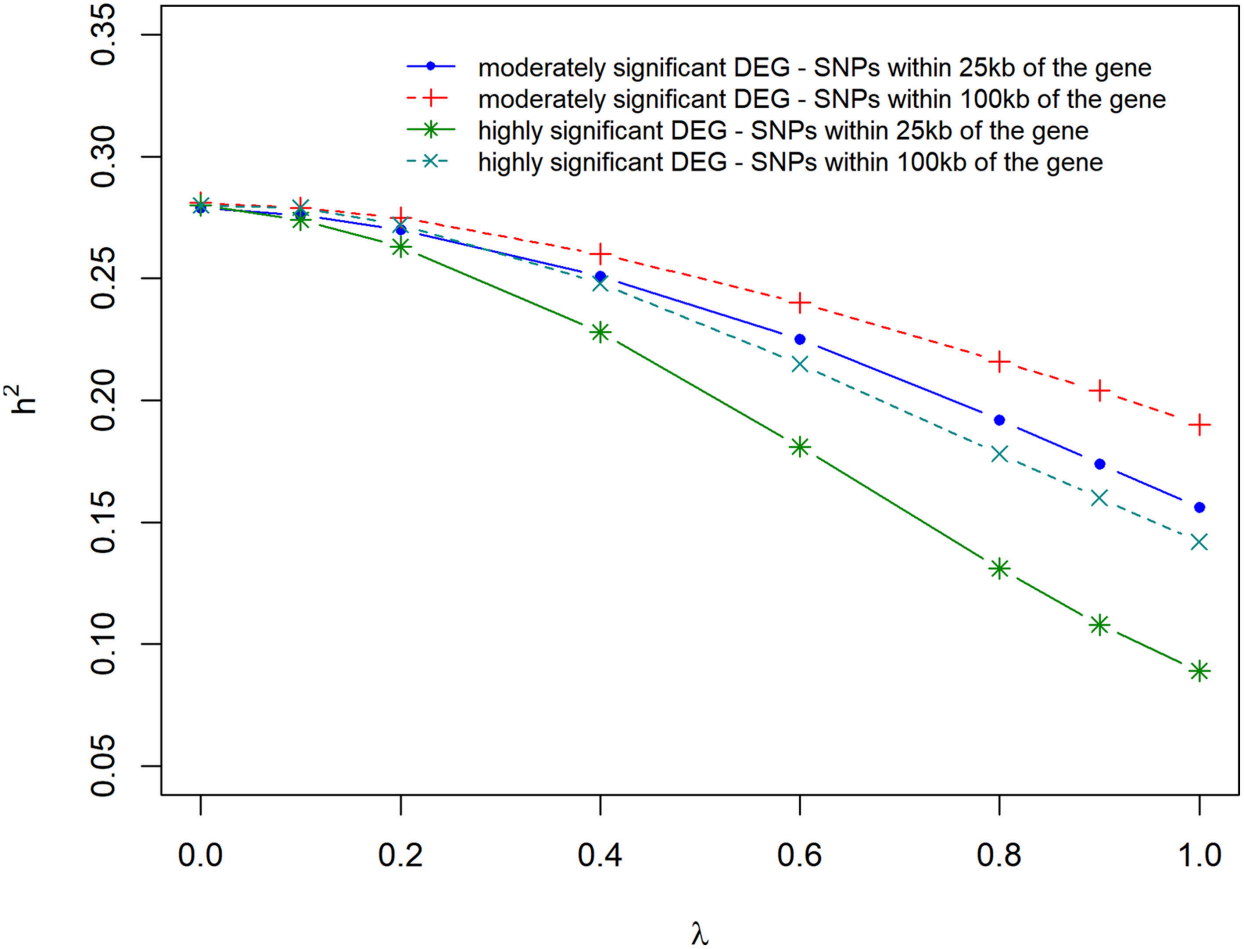
Estimate of heritability for different λ (blending of two genomic relationship matrices) values. The two genomic relationship matrices were generated for SNPs that were either within significant differentially expressed (DE) genes in gene expression analysis or not. Markers within significant DE genes were also weighted with the *log2*Δ*F* of that gene.

Figure 5 and Supplementary Table 1 present the results of the variance component and heritability estimates with different λ values. There was a gradual decrease in heritability when λ increased from 0 to 1. The reduction in heritability was different for the scenarios studied. When λ = 0, *h*^2^ was estimated to be 0.28 but decreased by 14% when using markers that were located within 100 kb region of moderately significant (*p* ≤ 2.4 × 10^−3^) DE genes. The highest loss (35%) in *h*^2^ was observed when using markers that were located within 25 kb region of highly significant (*p* ≤ 1.0 × 10^−4^) DE genes. Using markers that were located within 25 kb or 100 kb region of DE genes with *p* ≤ 2.4 × 10^−3^ and *p* ≤ 1.0 × 10^−4^, resulted in a similar loss (20%) in heritability estimate.

### Accuracy and Bias of Prediction

**Figure 6:**
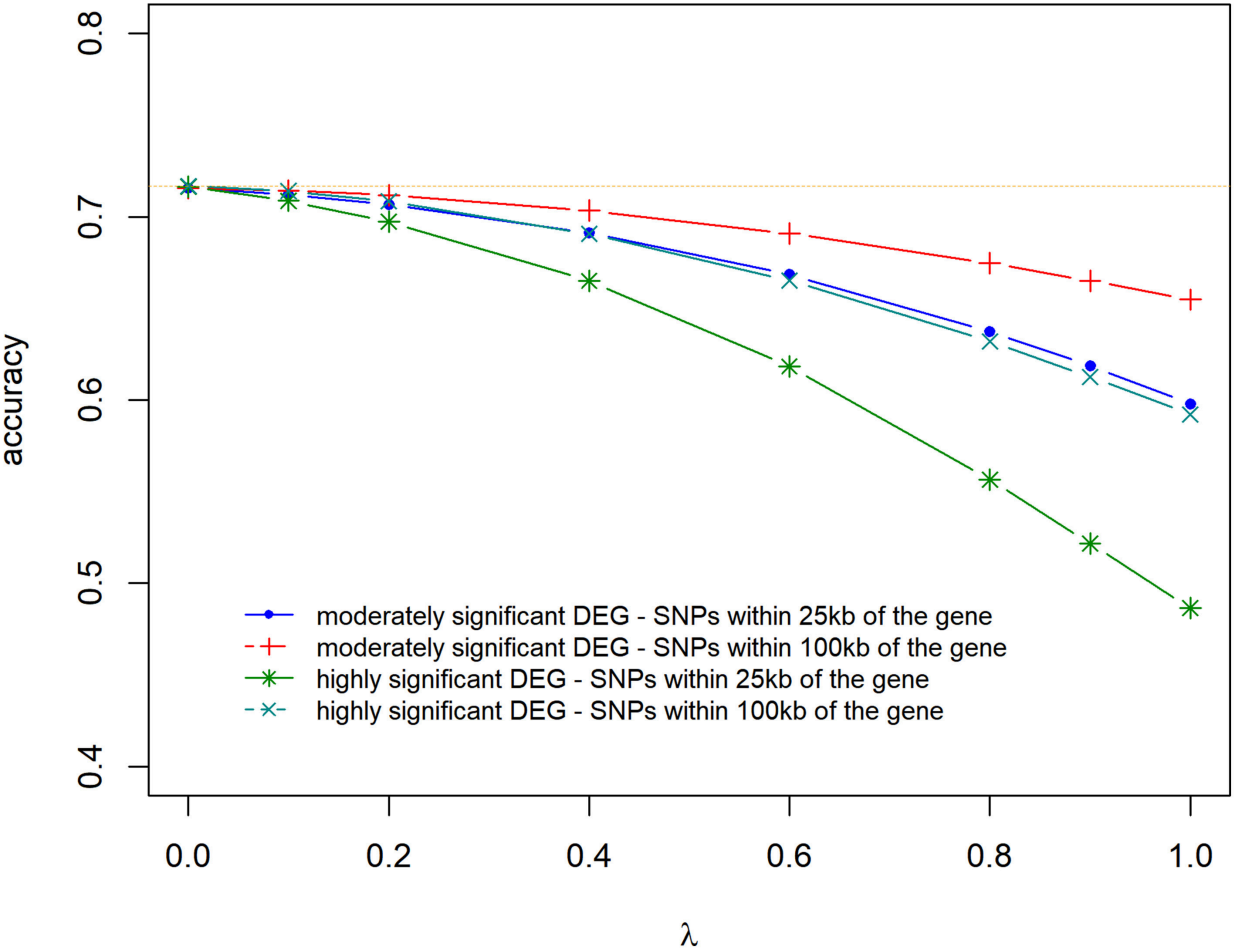
Accuracy of selection of genomic predictions for different λ (blending of two genomic relationship matrices) values. The two genomic relationship matrices were generated for SNPs that were either within significant genes in gene expression analysis or not. Markers within significant DE genes were also weighted with the *log*_2_Δ*F* of that gene.

**Figure 7:**
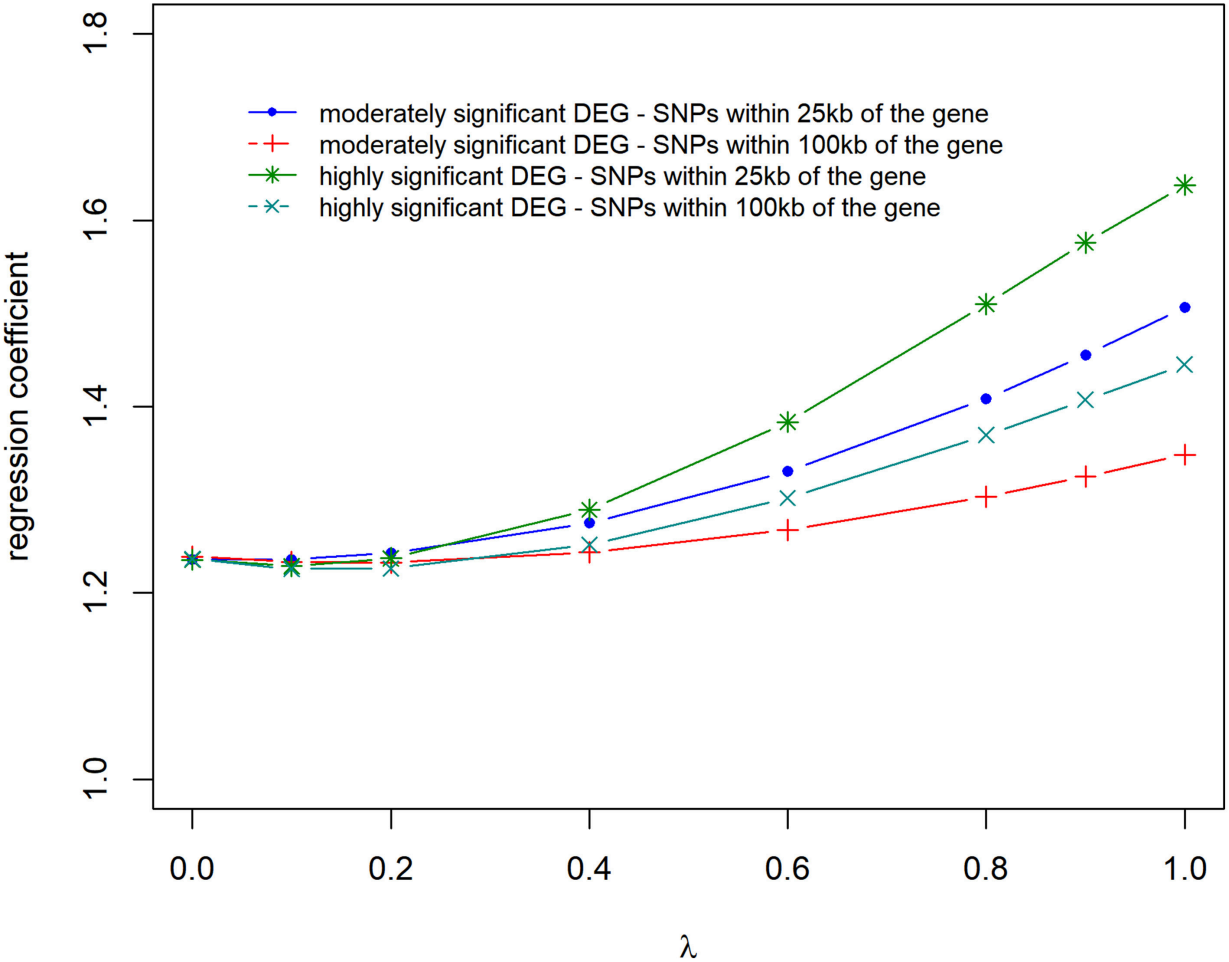
Regression coefficient of adjusted phenotype on genomic breeding values for different λ (blending of two genomic relationship matrices) values. The two genomic relationship matrices were generated for SNPs that were either within significant genes in gene expression analysis or not. Markers within significant DE genes were also weighted with the *log*_2_Δ*F* of that gene.

Prediction accuracies and biases are shown in Figures 6, 7, for all the 4 scenarios studied. Accuracies were highest, and biases were lowest when λ = 0 for all scenarios, thus using DE information and weighting SNPs based on the *log*_2_Δ*F* values decreased prediction accuracy and increased bias of breeding values (EBV). The maximum and minimum accuracy was 0.72 ± 0.06 and 0.49 ± 0.06, respectively. The loss in accuracy was <15% for all scenarios when λ ≤ 0.60 (Figure 6). When λ = 1 and *G* was constructed with markers within 100 kb region of moderately significant (*p* ≤ 2.4 × 10^−3^) DE genes, the decrease in accuracy was 9.30% compared to when *G* was constructed with markers within 25 kb, where the decrease in accuracy was the highest (47.08%). The same trend was observed for the scale of predictions of estimated EBVs. GEBVs were deflated even at λ = 0 (regression coefficient of 1.24 ± 0.23), and the incorporation of DE information (λ > 0) further increased the deflation of GEBVs.

Although one of the aims of this study was to uncover the potential of using results from gene expression analysis to increase the accuracy of genomic prediction, we also computed the prediction accuracy and bias with pedigree information. Accuracy and bias from pedigree-based analysis were 0.61 (SD = 0.07) and 1.30 (SD = 0.14), as compared to 0.72 (SD = 0.06) and 1.24 (SD = 0.23) for GEBVs.

## Discussion

Similar to some previous studies, our findings also confirm the higher levels of transcription in infected animals for some genes functioning in immune and inflammatory responses such as interleukin-1 beta, a pro-inflammatory cytokine (Bridle et al., 2006; Morrison et al., 2007) and CCAAT/enhancer binding protein beta (Wynne et al., 2008a). Interleukin-1 beta has been indicated as one of the hallmarks of Atlantic salmon response to AGD with its transcript showing a linear pattern of up-regulation throughout the disease progression starting from as early as day 12 post infection (Morrison et al., 2012; Nowak et al., 2014). Our data partially support this finding, as we found the expression in the naïve animals to be significantly higher compared to the animals during the first infection while the expression increased up to 3-folds during the second infection. We found no differences in transcriptional levels between animals with different severity of infection during the second infection stage (Supplementary Figure 6).

Also consistent with other studies (e.g., Wynne et al., 2008b; Young et al., 2008), we are reporting the down-regulation of some immune-related genes in AGD infected animals compared to the naïve fish (Figures 2A,B; Supplementary Figures 3a,b). The down-regulation of these genes is despite the finding that ~60% of differentially expressed transcripts had elevated their levels of expression in infected animals. Such an immunosuppression and down-regulation of different elements of host's immune system might be a means by which *N. perurans* restricts the host response and facilitates animal infection (Wynne et al., 2008a,b; Young et al., 2008; Benedicenti et al., 2015; Pennacchi et al., 2016). Similar patterns have also been reported in some other fish and human parasite infections (Sitjà-Bobadilla, 2008). However, previously it was suggested that the observed down-regulation of the immune genes is likely to represent an artifact of cell types, as the AGD-like lesions mainly consist of epithelial cells and these cells generally lack the majority of immune component signatures (Nowak et al., 2014; Pennacchi et al., 2014). Our data provide little support for this view as we found up-regulation of various immune genes during the second infection compared to the first infection and very little difference in the immune response between the animals at the second infection with different severity in gill lesions.

An interesting pattern that emerged from the analyses of whole transcriptome data is the identification of over-representation of transcripts, during the second infection or among more severely infected animals, with functional properties enriched in cell adhesion (Figure 3; Supplementary Figure 3c). These findings point to the potential importance of such molecules in AGD pathogenesis. The cell-cell interaction and adhesion is a fundamental step in a diverse array of physiological or pathological processes including angiogenesis, immune response and inflammation (González-Amaro, 2011). The up-regulation of transcripts belonging to some adhesion-related genes in more severely affected animals might be an indication of more intimate interference of amoeba with the host's cells in those infected fish and potentially a signature of the glycoproteins within the host mucus to interact more effectively with those of the *N. perurans*. It has been shown that at least some expressed host gene products that are involved in cell-cell adhesion, to also act as receptors for binding of pathogens to selective tissues (Soler et al., 2001). Therefore, it can be speculated that genetic variations within these genes to potentially be important in conferring higher tolerance against the amoeba. Identification of significant association of genetic markers on ssa04 and ssa09 which are in proximity to various members of the cadherin gene family provides further support for this view.

The heritability estimate in this study was similar to those reported by Robledo et al. (2018) and Taylor et al. (2009a) but higher than the estimate obtained by Gjerde et al. (2017). The higher heritability observed in this study compared to the study of Gjerde et al. (2017) could be due to the increased resolution of the scoring system in this study. Heritability estimate from first infection has been reported to be lower than those from the second or third infections (Taylor et al., 2009a; Kube et al., 2012). However, estimates based on a different SalmoBreed breeding nucleus year-class (Gjerde et al., 2017) suggests an opposite trend. In a simplistic scenario, we can say that response of the host organism to the first infections (challenge) might mainly trigger genes involved in the innate immunity, while the response to subsequent infections seems to stimulate more of the adaptive immune system genes.

The heritability estimate with genomic information in our study was also lower than the estimate from pedigree. The reduction in genetic variance observed while using genetic markers has also been previously reported by several authors (e.g., Erbe et al., 2013; Loberg et al., 2015; Robledo et al., 2018). Depending on the trait, heritability estimate from markers have been 10–50% lower than those with the pedigree information (Erbe et al., 2013; Loberg et al., 2015; Robledo et al., 2018). Factors such as incomplete LD between SNP variants and the causative mutations, marker density, and non-availability of other variations in the genome that might be associated with the trait can explain the reduction in heritability estimate with marker information (Erbe et al., 2013; de los Campos et al., 2015).

The results of the gene expression analysis of the AGD infection in a controlled challenge test environment were used to separate marker information into two groups: i. Markers within genes that were significant in the DE analysis and ii. Polygenic effect. Two genomic relationship matrices were constructed from the two marker sets and used directly in a GBLUP model to estimate genetic variation and EBVs. Further, we applied differential weights (based on *log*_2_Δ*F* values) to the markers that were located within/close-to significant genes identified from the DE analysis. An advantage of incorporating results from DE-based analysis through weighted genomic relationship matrix approach is that it can be easily incorporated into genetic evaluation routines.

In this study, we observed that the genetic variance, heritability, accuracy and bias of genomic prediction decreased when DE information was used. de Los Campos et al. (2013) also observed a reduced heritability (2.75–28%) when using a weighted genomic relationship matrix for variance component estimation. Heritability reduced by 0–50% in this study and was dependent on the 1 − λ (polygenic markers) parameter. The loss in genetic variance did not, however, decrease genomic prediction accuracy in the study of de Los Campos et al. (2013). Su et al. (2014) also observed an increase in accuracy using weighted genomic relationship matrix with −*log*_10_*p*-value weights from a GWAS. However, the opposite was observed by Ni et al. (2017). The reason for the decline in prediction accuracy in this study could be because of the uncertainty and noise (sampling variance) of the estimated *log*_2_Δ*F* from the DE analysis. Since in most gene expression analysis, the estimate of this effect is based on only a few samples, sampling variance of these estimates are often large and can affect the predictive accuracy of genomic prediction.

Furthermore, the decline in prediction accuracy could also be attributed to using DE results from a different year-class/population to give differential weights to markers. There is some level of genetic differentiation between these populations since they came from a different genetic base, and therefore the response to AGD in these two population might be different. We also used −*log*_10_ *p* as differential weight, but the trend we observed for accuracy, bias, and heritability was the same as using the *log*_2_Δ*F*, except that the reduction was lower with the −*log*_10_ *p*-values (Supplementary Figures 7a,b).

In this study, the use of genomic information resulted in about 18% increase in prediction accuracy and 4.8% decrease in the bias of EBVs compared to using pedigree information. Recently, Robledo et al. (2018) reported an increase of 18% in prediction accuracy and 12.5% reduction in the bias of EBVs for resistance to AGD from using genomic information compared to pedigree information. Several other studies have also pointed out the potential of using genomic information in aquaculture species for parasite resistance (e.g., Odegård et al., 2014; Correa et al., 2017).

In conclusion, our transcriptomic analysis of fish during naïve and subsequent infections with *N. perurans* has now provided a more detailed profile of the host's response to this amoeba. The results presented in this study have indicated changes in expression of many genes, particularly genes with functional properties in the immune system as well as in cellular-adhesion among infected animals. While during the initial stages of the infection many components of the immune system seem to have significantly reduced their levels of expression, an increase in the transcription of some immune genes is reported during the second challenge. Further, comparative analysis of animals with different severity to infection highlights the potential importance of genes such as perforin-1, ENPP2 and CCL20 in conferring higher resistance against AGD. The up-regulation of many genes involved in cell adhesion processes provides further clues to AGD pathogenesis. Some of these genes are in proximity to the QTL identified through GWAS, further supporting their potential role in resistance against this disease. With regards to prediction accuracy and bias of EBVs, the results from this study show that accuracy of selection and bias EBVs were better with genomic than pedigree information. However, using information from gene expression analysis to further increase the accuracy of genomic predictions through blending of different genomic relationship matrices in a GBLUP model was not beneficial regardless of the weighting factors (*log*_2_Δ*F*or −*log*_10_*p*) and λ (the amount of blending of G matrices used).

## Author Contributions

BG conceived and designed the experiment. BG and BH organized and supervised the execution of the challenge tests and sample collections. HM and SM-N performed RNA extraction, quantification, bioinformatics and statistical analysis related to the transcriptomic data. SB performed analysis of the genomic data. HM and SB drafted the manuscript. All authors contributed to the development of the manuscript and interpretation of the data.

### Conflict of Interest Statement

The authors declare that the research was conducted in the absence of any commercial or financial relationships that could be construed as a potential conflict of interest.

## Supplementary Material

Supplementary Table 1 Estimate of variance component when using different information sources to build the genomic relationship matrix.

Supplementary Figure 1 Number and distribution of animals with different AGD scores, for whole transcriptome sequencing during the 3 sampling stages, naïve, first and the second infections. The animals were selected from the challenge test conducted during 2015.

Supplementary Figure 2 A Venn diagram, showing the overlaps of the differentially expressed transcripts between the samples collected at the naïve (n), first (i1), and the second infections (i2). The total number of differentially expressed genes have been indicated in the brackets.

Supplementary Figure 3 Bar graphs showing the top functional categories in the gene ontology domain of biological processes. The pathways are indicated on the y-axis, while the x-axis depicts the significant scores (–*log*_10_*p* value calculated based on Fisher exact test). **(a)** Naïve vs. primary infection of down-regulated genes; **(b)** Naïve vs. secondary infection of down-regulated genes; **(c)** Naïve vs. secondary infection of up-regulated genes; **(d)** Naïve vs. secondary infection of up-regulated immune genes.

Supplementary Figure 4 Distribution of AGD scores of the 2016 year-class of the SalmoBreed population. AGD scores were based on the sum of Taylor et al. (2009a,b) score of all 16 gill surfaces of infected animals.

Supplementary Figure 5 Quantile-quantile plot for the test statistics used in the genome-wide association analysis of resistance to AGD.

Supplementary Figure 6 Expression bar plot of interleukin-1 beta, shown in FPKM, and the associated standard errors for the 36 sequenced animals at the naïve stage (n), first infection (i1), second infection with score 2 (i2_2) or the second infection with score 3 (i2_3).

Supplementary Figure 7 **(a)** Accuracy of selection of genomic predictions and **(b)** regression coefficient of adjusted phenotype on genomic breeding values for different λ (blending of two genomic relationship matrices) values. The two genomic relationship matrices were generated for SNPs that were either within significant genes in gene expression analysis or not. Markers within significant DE genes were also weighted with the –*log*_*10*_*p* of that gene.
