# Supplementary material for: Genomic and Transcriptomic Analysis of Amoebic Gill Disease Resistance in Atlantic Salmon (Salmo salar L.)
Source: Front Genet. 2019 Feb 27;10:68. doi: 10.3389/fgene.2019.00068 (PMC6400892; doi:10.3389/fgene.2019.00068)
Supplement: Supplementary Table 1 — Estimate of variance component when using different information sources to build the genomic relationship matrix. [file Table_1.DOCX]

**Supplementary Table 1:** Estimate of variance component when using different information sources to build the genomic relationship matrix.

| Information  Source | $\lambda$ | Within 100kbs of a DE gene | | | | | |  | Within 25kbs of a DE gene | | | | | |
| --- | --- | --- | --- | --- | --- | --- | --- | --- | --- | --- | --- | --- | --- | --- |
|  |  | $\sigma_{g}^{2}$ | $\sigma_{c}^{2}$ | $\sigma_{e}^{2}$ | $\sigma_{p}^{2}$ | $h^{2}$ | $c^{2}$ |  | $\sigma_{g}^{2}$ | $\sigma_{c}^{2}$ | $\sigma_{e}^{2}$ | $\sigma_{p}^{2}$ | $h^{2}$ | $c^{2}$ |
| Highly significant DE genes | 0.10 | 3.863 (0.81) | 0.625 (0.391) | 9.182 (0.505) | 13.670 (0.628) | 0.283 (0.053) | 0.046 (0.028) |  | 3.834 (0.806) | 0.637 (0.391) | 9.194 (0.505) | 13.665 (0.627) | 0.281 (0.053) | 0.047 (0.028) |
|  | 0.20 | 3.846 (0.806) | 0.631 (0.389) | 9.189 (0.505) | 13.666 (0.627) | 0.281 (0.053) | 0.046 (0.028) |  | 3.821 (0.803) | 0.642 (0.389) | 9.202 (0.503) | 13.665 (0.627) | 0.280 (0.052) | 0.047 (0.028) |
|  | 0.40 | 3.765 (0.794) | 0.664 (0.391) | 9.227 (0.503) | 13.656 (0.626) | 0.276 (0.052) | 0.049 (0.028) |  | 3.719 (0.788) | 0.687 (0.393) | 9.257 (0.500) | 13.662 (0.626) | 0.272 (0.052) | 0.050 (0.028) |
|  | 0.60 | 3.631 (0.776) | 0.722 (0.392) | 9.293 (0.499) | 13.646 (0.624) | 0.266 (0.051) | 0.053 (0.028) |  | 3.535 (0.763) | 0.771 (0.395) | 9.351 (0.495) | 13.657 (0.624) | 0.259 (0.050) | 0.056 (0.029) |
|  | 0.80 | 3.458 (0.753) | 0.799 (0.396) | 9.379 (0.494) | 13.637 (0.622) | 0.254 (0.050) | 0.059 (0.029) |  | 3.291 (0.731) | 0.885 (0.399) | 9.473 (0.489) | 13.649 (0.622) | 0.241 (0.049) | 0.065 (0.029) |
|  | 0.90 | 3.360 (0.739) | 0.844 (0.396) | 9.429 (0.491) | 13.633 (0.621) | 0.246 (0.049) | 0.062 (0.029) |  | 3.152 (0.713) | 0.951 (0.401) | 9.542 (0.486) | 13.645 (0.621) | 0.231 (0.048) | 0.070 (0.029) |
|  | 1.00 | 3.257 (0.724) | 0.892 (0.398) | 9.480 (0.489) | 13.630 (0.620) | 0.239 (0.048) | 0.066 (0.029) |  | 3.008 (0.693) | 1.021 (0.405) | 9.612 (0.483) | 13.642 (0.620) | 0.220 (0.046) | 0.075 (0.029) |
| Moderately significant DE genes | 0.10 | 3.857 (0.807) | 0.621 (0.388) | 9.187 (0.505) | 13.665 (0.627) | 0.282 (0.053) | 0.046 (0.028) |  | 3.823 (0.805) | 0.644 (0.39) | 9.199 (0.504) | 13.666 (0.627) | 0.280 (0.053) | 0.047 (0.028) |
|  | 0.20 | 3.833 (0.805) | 0.641 (0.391) | 9.195 (0.504) | 13.669 (0.628) | 0.280 (0.053) | 0.047 (0.028) |  | 3.770 (0.799) | 0.673 (0.392) | 9.225 (0.504) | 13.669 (0.627) | 0.276 (0.052) | 0.049 (0.028) |
|  | 0.40 | 3.724 (0.792) | 0.706 (0.395) | 9.245 (0.502) | 13.675 (0.628) | 0.272 (0.052) | 0.052 (0.029) |  | 3.557 (0.775) | 0.778 (0.397) | 9.331 (0.499) | 13.666 (0.626) | 0.260 (0.051) | 0.057 (0.029) |
|  | 0.60 | 3.547 (0.771) | 0.801 (0.398) | 9.331 (0.498) | 13.679 (0.627) | 0.259 (0.051) | 0.058 (0.029) |  | 3.242 (0.737) | 0.930 (0.406) | 9.488 (0.493) | 13.660 (0.623) | 0.237 (0.049) | 0.068 (0.029) |
|  | 0.80 | 3.318 (0.742) | 0.919 (0.405) | 9.444 (0.493) | 13.681 (0.626) | 0.242 (0.049) | 0.067 (0.029) |  | 2.867 (0.688) | 1.111 (0.413) | 9.673 (0.486) | 13.652 (0.620) | 0.210 (0.046) | 0.081 (0.030) |
|  | 0.90 | 3.188 (0.726) | 0.986 (0.407) | 9.508 (0.490) | 13.682 (0.626) | 0.233 (0.048) | 0.072 (0.029) |  | 2.668 (0.660) | 1.209 (0.417) | 9.770 (0.482) | 13.647 (0.619) | 0.196 (0.045) | 0.089 (0.030) |
|  | 1.00 | 3.051 (0.706) | 1.055 (0.411) | 9.576 (0.487) | 13.682 (0.625) | 0.223 (0.047) | 0.077 (0.029) |  | 2.470 (0.632) | 1.307 (0.421) | 9.866 (0.478) | 13.643 (0.618) | 0.181 (0.043) | 0.096 (0.030) |

$\sigma_{g}^{2}$= genetic variance;$\sigma_{c}^{2}$= common environmental variance; $\sigma_{e}^{2}$= residual variance; $\sigma_{p}^{2}$= phenotypic variance; $h^{2}$= heritability
