# Supplementary figures and images for: Genomic and Transcriptomic Analysis of Amoebic Gill Disease Resistance in Atlantic Salmon (Salmo salar L.)
Source: Front Genet. 2019 Feb 27;10:68. doi: 10.3389/fgene.2019.00068 (PMC6400892; doi:10.3389/fgene.2019.00068)

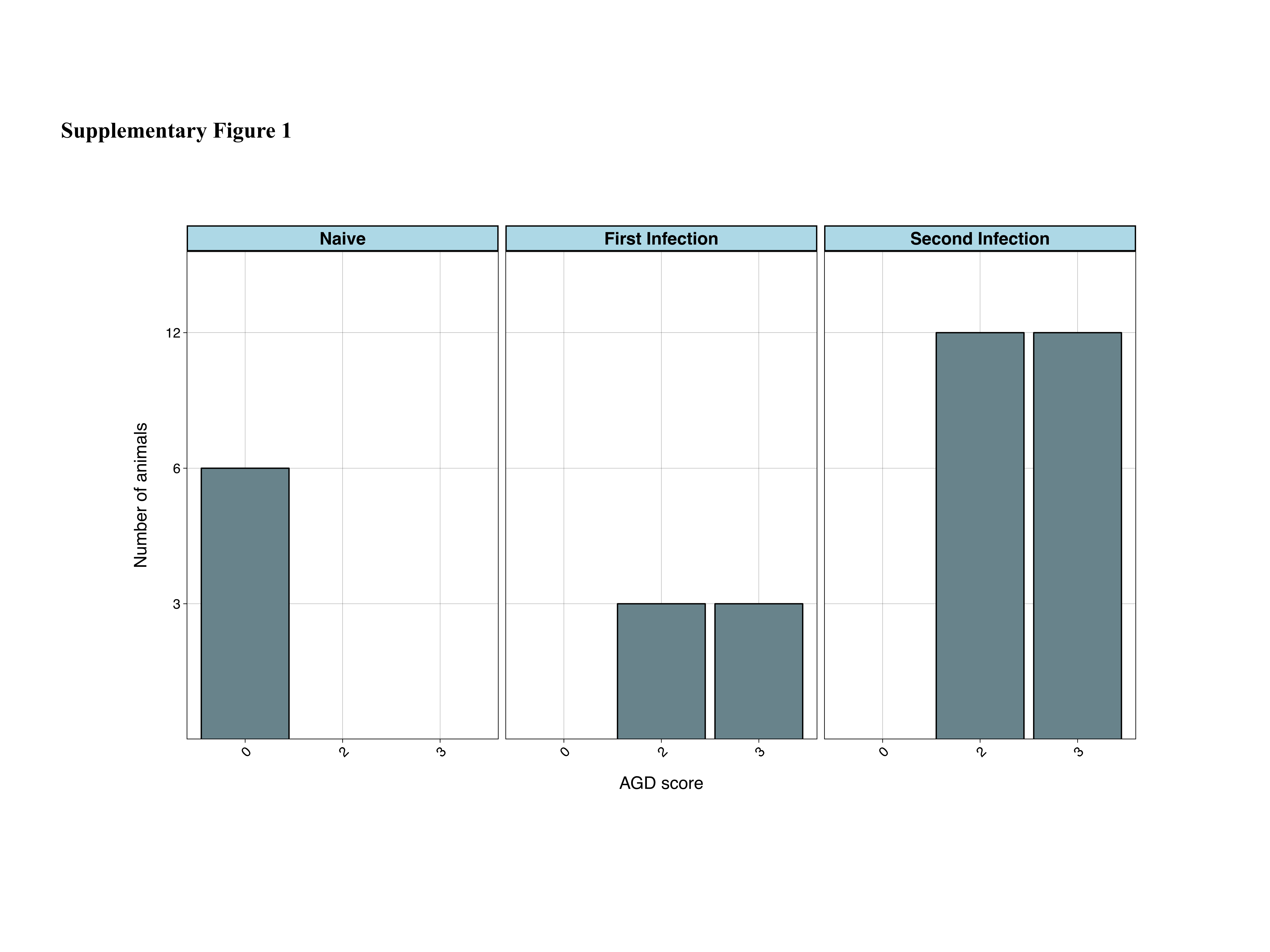

Supplement: Supplementary Figure 1 — Number and distribution of animals with different AGD scores, for whole transcriptome sequencing during the 3 sampling stages, naïve, first and the second infections. The animals were selected from the challenge test conducted during 2015. [file Image_1.TIFF]

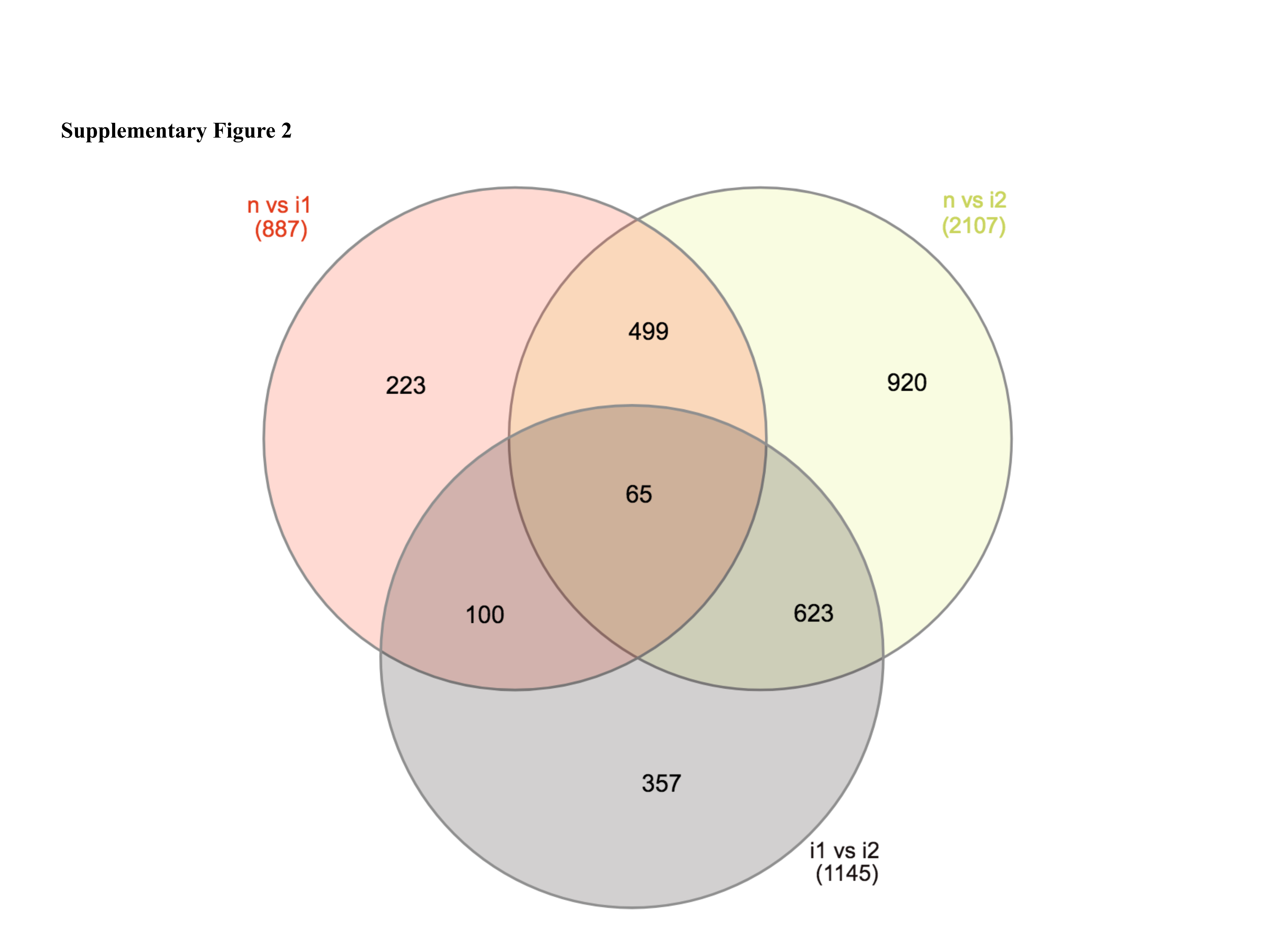

Supplement: Supplementary Figure 2 — A Venn diagram, showing the overlaps of the differentially expressed transcripts between the samples collected at the naïve (n), first (i1), and the second infections (i2). The total number of differentially expressed genes have been indicated in the brackets. [file Image_2.TIFF]

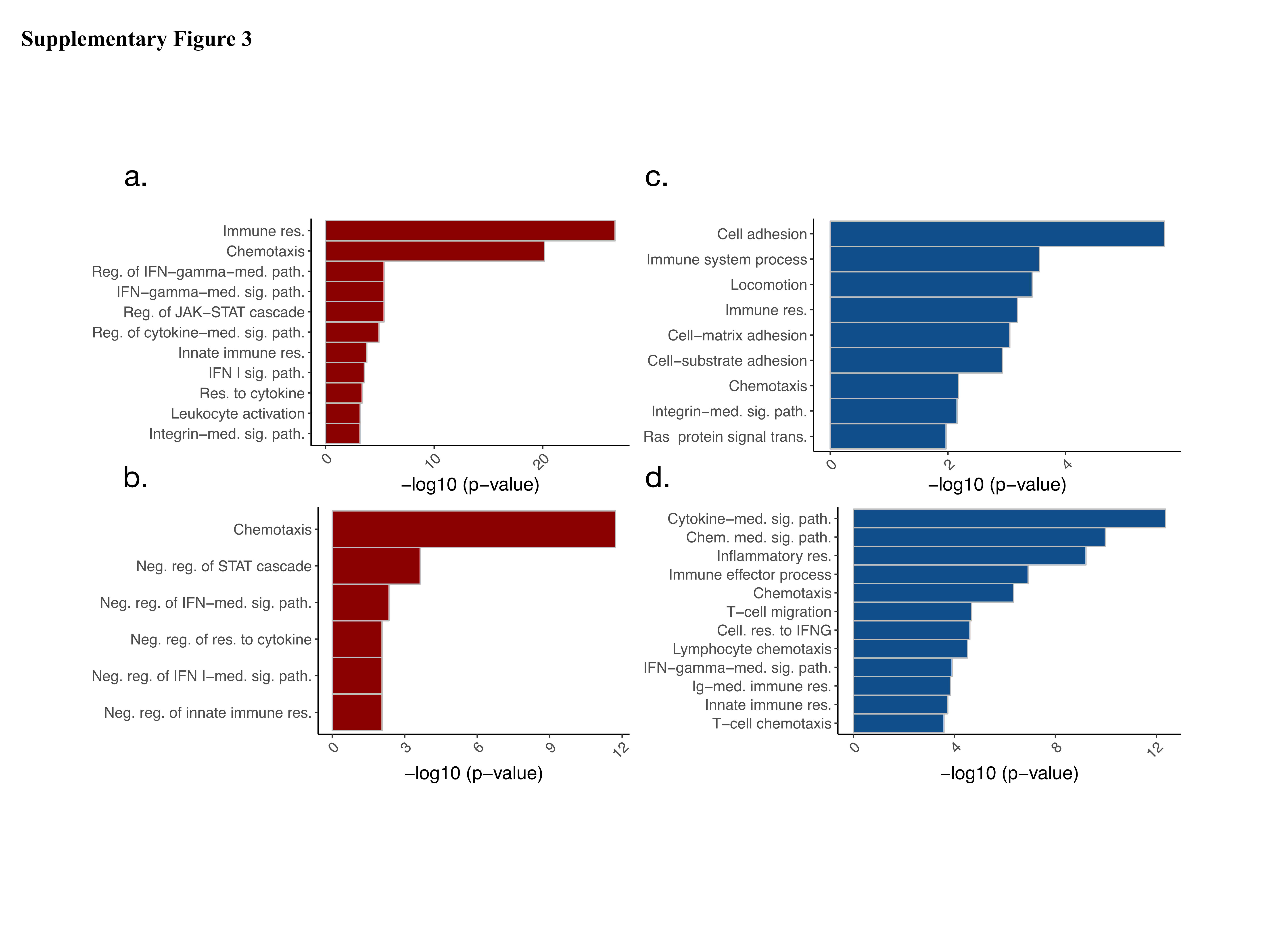

Supplement: Supplementary Figure 3 — Bar graphs showing the top functional categories in the gene ontology domain of biological processes. The pathways are indicated on the y-axis, while the x-axis depicts the significant scores (–log10p value calculated based on Fisher exact test). (a) Naïve vs. primary infection of down-regulated genes; (b) Naïve vs. secondary infection of down-regulated genes; (c) Naïve vs. secondary infection of up-regulated genes; (d) Naïve vs. secondary infection of up-regulated immune genes. [file Image_3.TIFF]

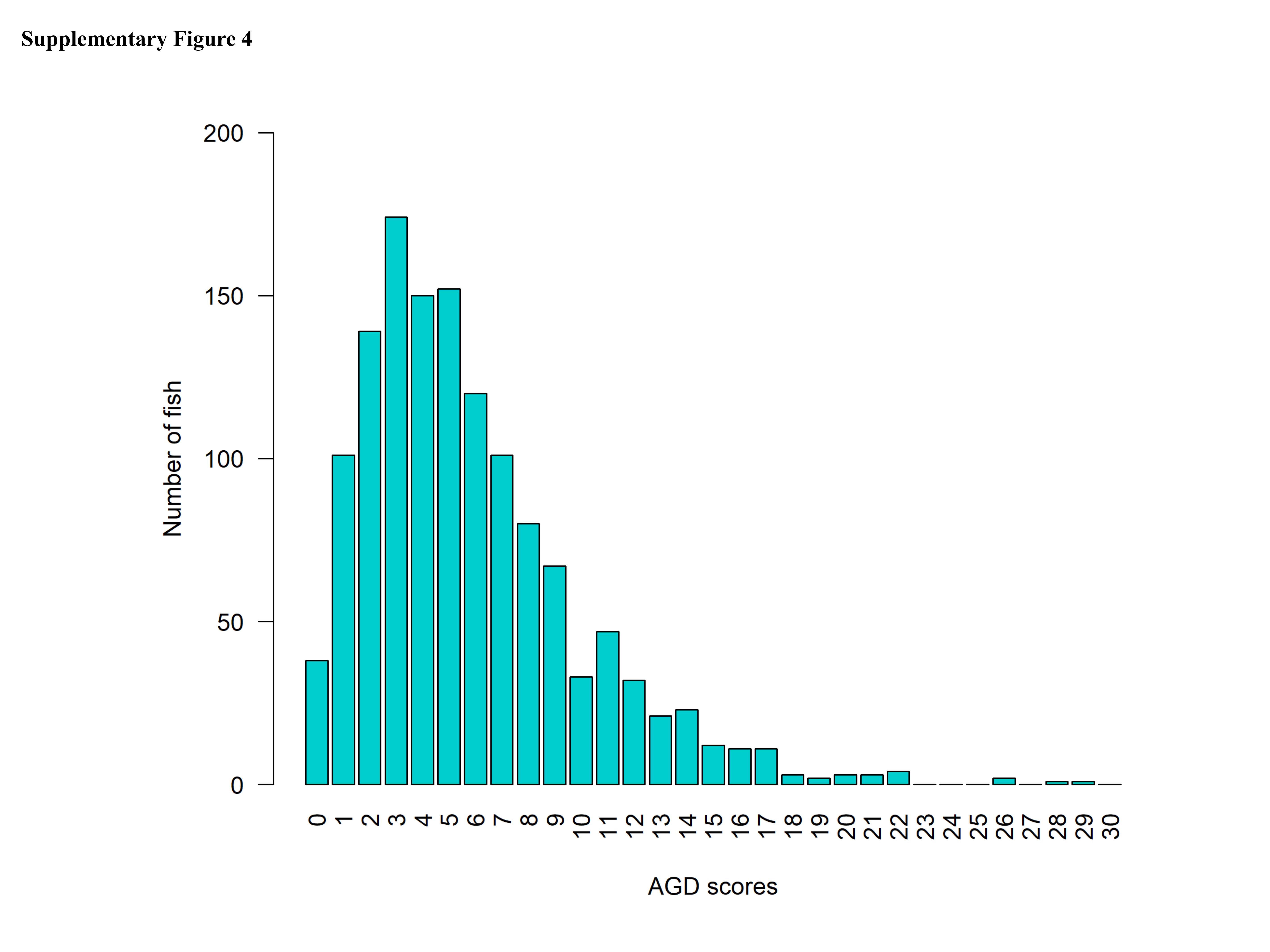

Supplement: Supplementary Figure 4 — Distribution of AGD scores of the 2016 year-class of the SalmoBreed population. AGD scores were based on the sum of Taylor et al. (2009a,b) score of all 16 gill surfaces of infected animals. [file Image_4.TIFF]

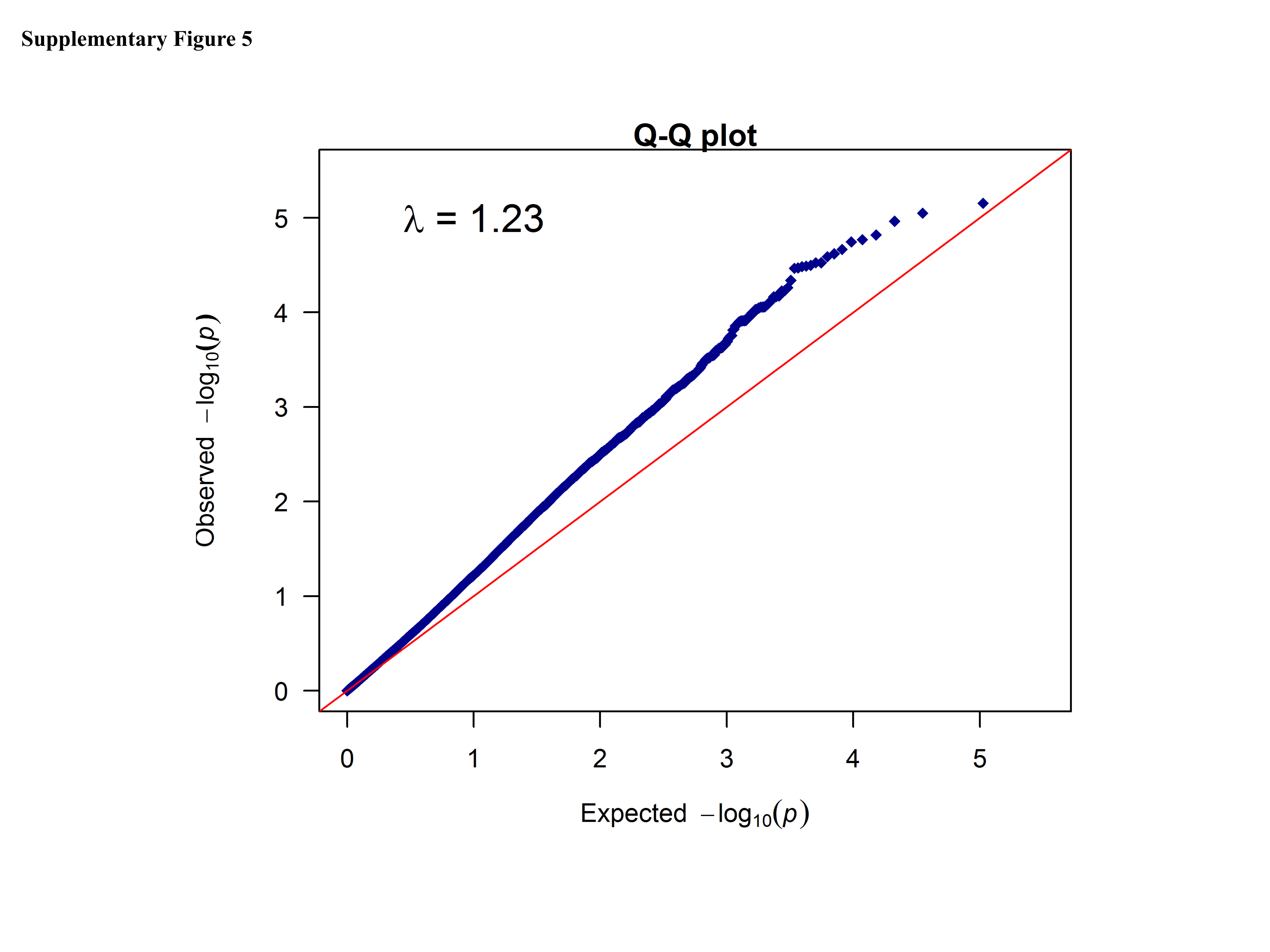

Supplement: Supplementary Figure 5 — Quantile-quantile plot for the test statistics used in the genome-wide association analysis of resistance to AGD. [file Image_5.TIFF]

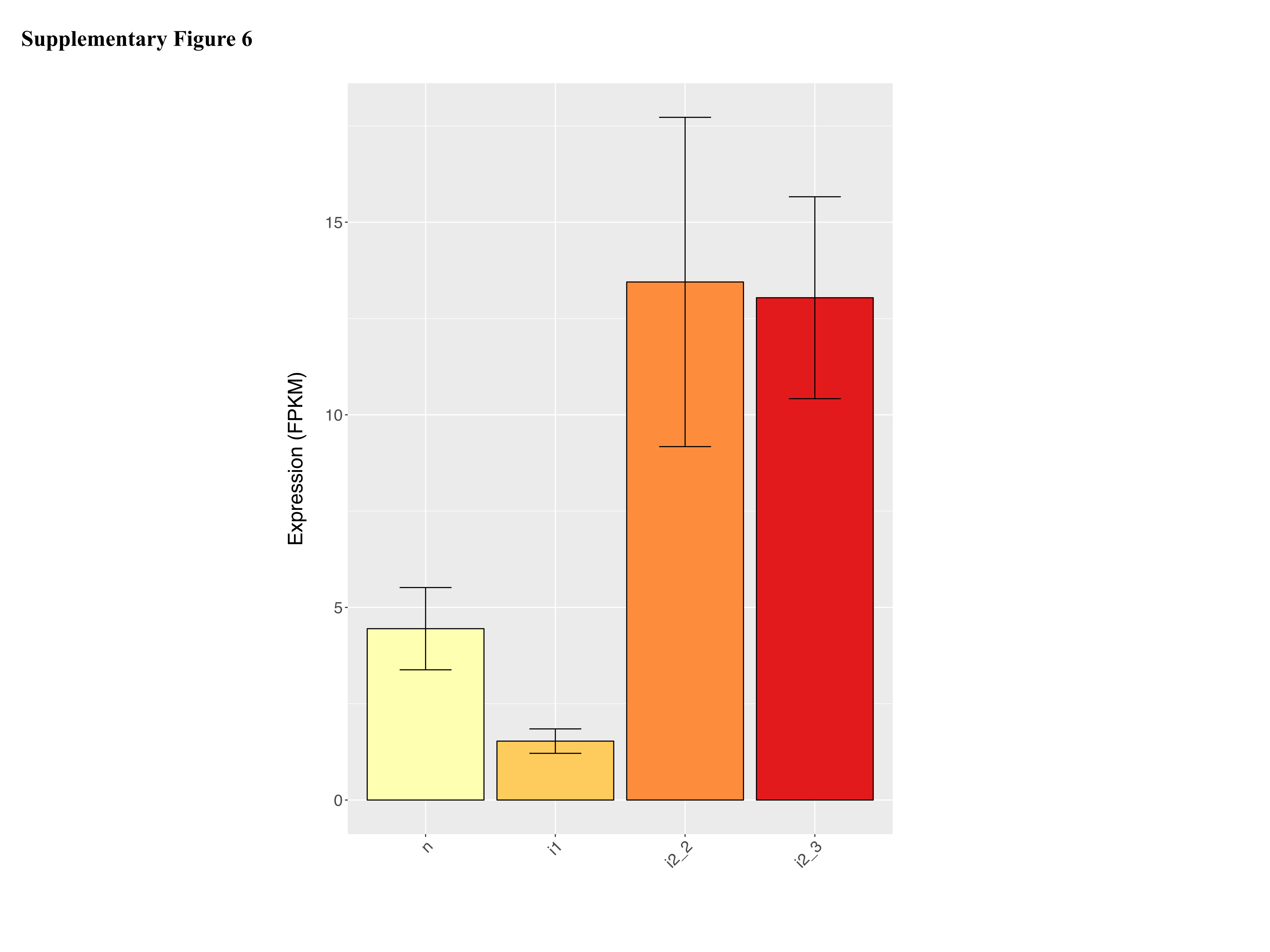

Supplement: Supplementary Figure 6 — Expression bar plot of interleukin-1 beta, shown in FPKM, and the associated standard errors for the 36 sequenced animals at the naïve stage (n), first infection (i1), second infection with score 2 (i2_2) or the second infection with score 3 (i2_3). [file Image_6.TIFF]

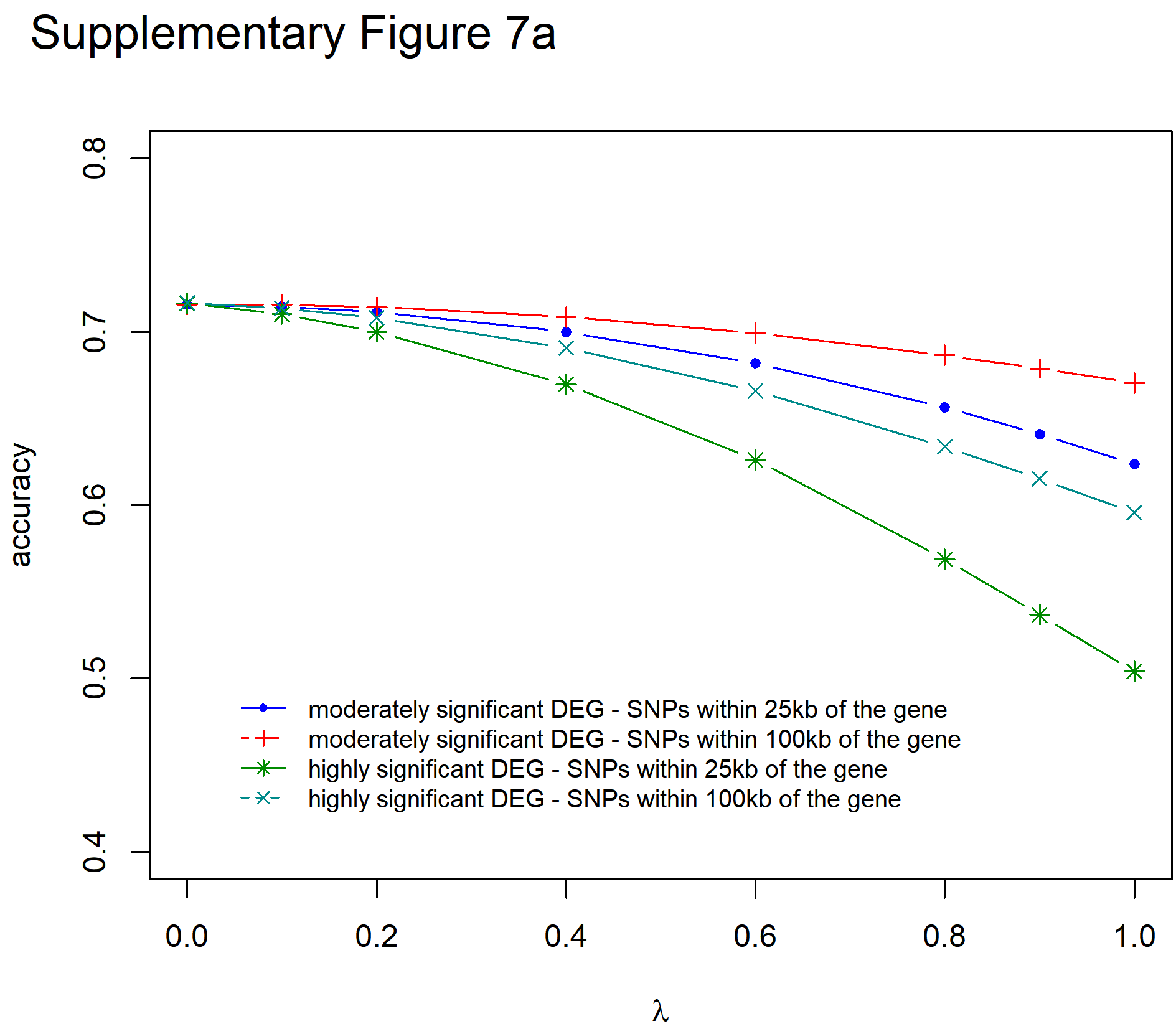

Supplement: Supplementary Figure 7 — (a) Accuracy of selection of genomic predictions and (b) regression coefficient of adjusted phenotype on genomic breeding values for different λ (blending of two genomic relationship matrices) values. The two genomic relationship matrices were generated for SNPs that were either within significant genes in gene expression analysis or not. Markers within significant DE genes were also weighted with the –log10p of that gene. [file Image_7.TIFF]

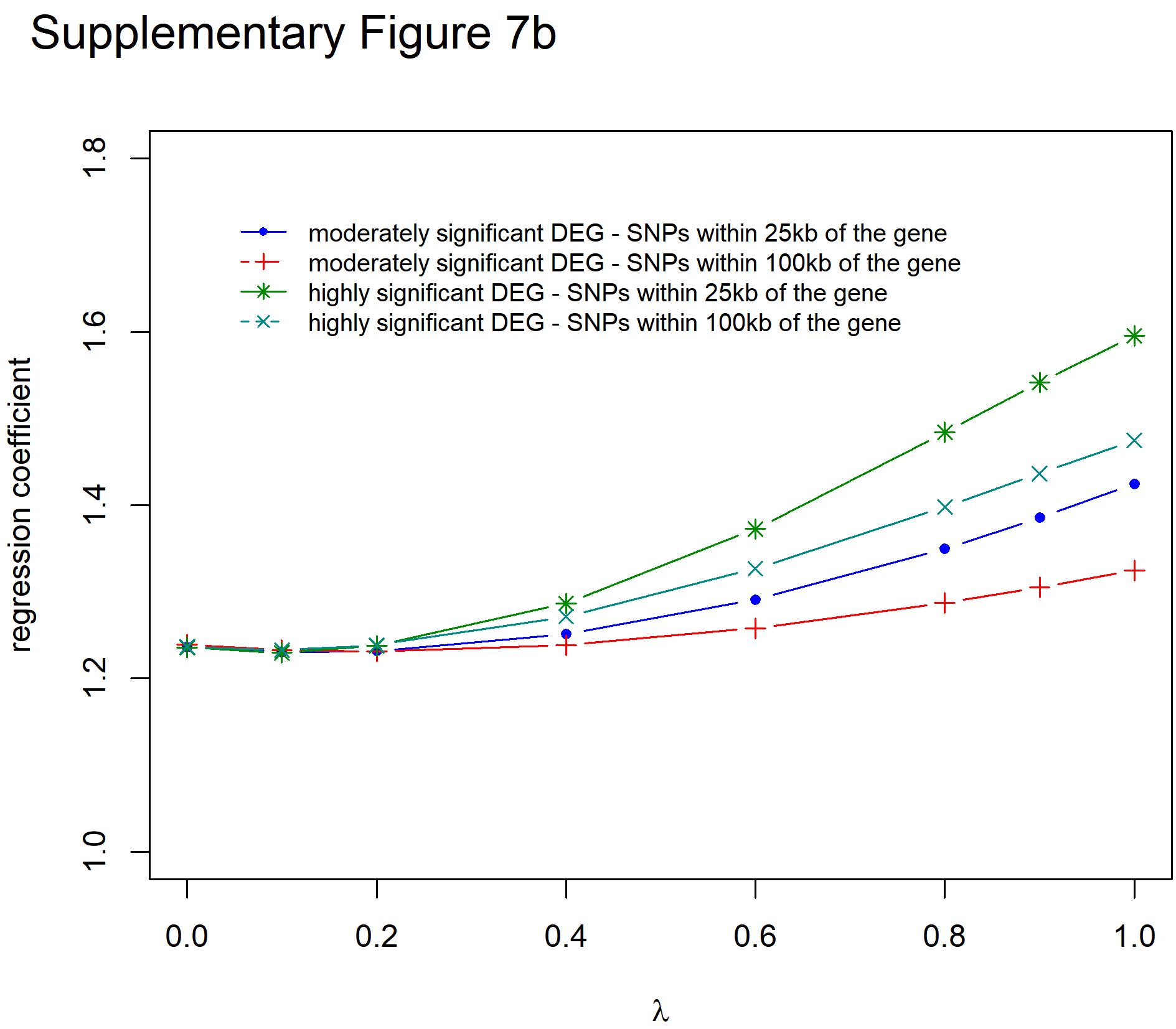

Supplement: Supplementary file 9 [file Image_8.TIFF]
